# Postnatal Changes in the Expression Pattern of the Imprinted Signalling Protein XLαs Underlie the Changing Phenotype of Deficient Mice

**DOI:** 10.1371/journal.pone.0029753

**Published:** 2012-01-11

**Authors:** Stefan O. Krechowec, Katie L. Burton, Anna U. Newlaczyl, Nicolas Nunn, Nikolina Vlatković, Antonius Plagge

**Affiliations:** 1 Department of Cellular and Molecular Physiology, Institute of Translational Medicine, University of Liverpool, Liverpool, United Kingdom; 2 Molecular and Clinical Cancer Medicine, Institute of Translational Medicine, University of Liverpool, Liverpool, United Kingdom; CNRS, France

## Abstract

The alternatively spliced trimeric G-protein subunit XLαs, which is involved in cAMP signalling, is encoded by the *Gnasxl* transcript of the imprinted *Gnas* locus. XLαs deficient mice show neonatal feeding problems, leanness, inertia and a high mortality rate. Mutants that survive to weaning age develop into healthy and fertile adults, which remain lean despite elevated food intake. The adult metabolic phenotype can be attributed to increased energy expenditure, which appears to be caused by elevated sympathetic nervous system activity. To better understand the changing phenotype of *Gnasxl* deficient mice, we compared XLαs expression in neonatal versus adult tissues, analysed its co-localisation with neural markers and characterised changes in the nutrient-sensing mTOR1-S6K pathway in the hypothalamus. Using a newly generated conditional *Gnasxl* lacZ gene trap line and immunohistochemistry we identified various types of muscle, including smooth muscle cells of blood vessels, as the major peripheral sites of expression in neonates. Expression in all muscle tissues was silenced in adults. While *Gnasxl* expression in the central nervous system was also developmentally silenced in some midbrain nuclei, it was upregulated in the preoptic area, the medial amygdala, several hypothalamic nuclei (e.g. arcuate, dorsomedial, lateral and paraventricular nuclei) and the nucleus of the solitary tract. Furthermore, expression was detected in the ventral medulla as well as in motoneurons and a subset of sympathetic preganglionic neurons of the spinal cord. In the arcuate nucleus of *Gnasxl*-deficient mice we found reduced activity of the nutrient sensing mTOR1-S6K signalling pathway, which concurs with their metabolic status. The expression in these brain regions and the hypermetabolic phenotype of adult *Gnasxl*-deficient mice imply an inhibitory function of XLαs in energy expenditure and sympathetic outflow. By contrast, the neonatal phenotype of mutant mice appears to be due to a transient role of XLαs in muscle tissues.

## Introduction

In mammals the paternally and maternally inherited genomes contribute unequally to the development of offspring [1], [2], which can be attributed to epigenetic modifications that are established in the two parental germlines. This process of ‘genomic imprinting’ results in the differential silencing and parent-of-origin specific, monoallelic expression of a subset of genes. [3]–[5] (for imprinted gene databases see: http://www.mousebook.org/catalog.php?catalog=imprinting and http://igc.otago.ac.nz). Many imprinted genes have been shown to influence embryonic growth, but an increasing number are now being recognised for their roles in postnatal development as well as having lasting effects on adult physiology and/or behaviour [6]–[12]. A recurring theme for the action of imprinted genes through embryonic and postnatal development is the partitioning of nutrients and other resources between mother and offspring [6], [7]. Furthermore, those imprinted genes that have been shown to exert a (continued) function at the adult stage usually impact on energy homeostasis or behaviour through their expression in metabolically relevant tissues and defined regions of the central nervous system (CNS) [6], [9], [12]. Many, but not all, murine imprinted gene functions are conserved in humans, and several human genetic disorders are associated with imprinted gene loci, e.g. Beckwith-Wiedemann-Syndrome, Prader-Willi-Syndrome/Angelman-Syndrome, Silver-Russell-Syndrome and Transient Neonatal Diabetes Mellitus [13].

**Figure 1:**
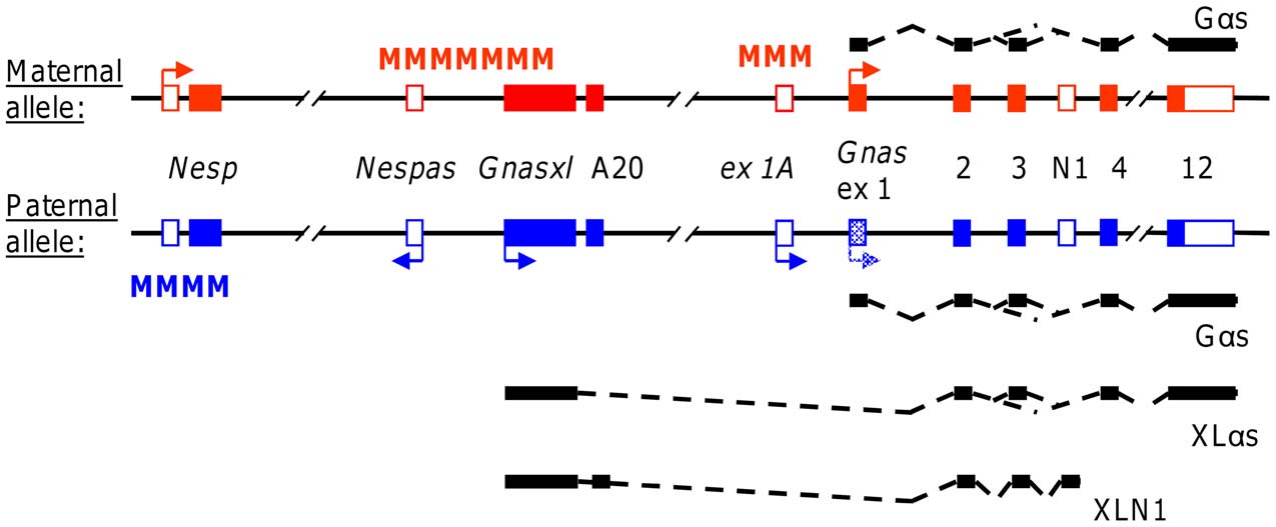
Simplified scheme of the imprinted murine *Gnas* locus. The differential, parental allele-specific expression of the various transcripts of the locus is indicated by arrows. Open and filled boxes represent non-coding and coding exons, respectively. Spliced transcripts and encoded proteins are only shown for *Gnas* and *Gnasxl* above and below the parental alleles. On the paternal allele *Gnas* expression is silenced in specific tissues (shaded *Gnas* exon 1 box). *Gnasxl* splices in frame onto exon 2 and encodes an ‘extra large’ α-subunit. Splicing onto exon N1 is limited to neural tissue, results in premature termination of the open reading frame and expression of a truncated XLN1 protein. The alternatively spliced exon A20 is found in a minority of *Gnasxl* transcripts, mainly in combination with exon N1, and introduces a frameshift and early termination codon in exon 2. *Nespas* and *exon 1A* promoters generate non-coding regulatory RNAs. The *Nesp* transcript splices onto exon 2 and has a regulatory as well as protein coding function. Regions of differential DNA methylation, including the imprinting control region (ICR) at *Nespas*, are symbolized by M.

One of the first genomic regions that was recognised as being imprinted is located on mouse distal chromosome 2 [14]. The imprinted locus responsible for the observed abnormal phenotypes was later identified as the *Gnas* locus, which is conserved on human chromosome 20q13.32 and consists of a complex arrangement of alternatively spliced transcripts and imprinted promoters that cause parent-of-origin specific effects when mutated (Figure 1) [15]–[20]. The two main protein-coding transcripts of the locus comprise *Gnas* itself, which codes for the cAMP stimulatory signalling protein Gα_s_, and its variant *Gnasxl*, which translates into XLαs, an NH_2_-terminally extended version of Gα_s_ [21]. *Gnas* and *Gnasxl* are derived from separate promoters, but share their open reading frame from exon 2 onwards. Despite their different NH_2_-termini, both proteins can stimulate adenylyl cyclase and cAMP production upon activation of various receptors in transfected cells [22]. However, the *Gnas* and *Gnasxl* transcripts differ with regard to their regulation by genomic imprinting. In most cell types *Gnas* is not imprinted, but in a subset of tissues its paternal allele is silenced, resulting in a reduced dosage of Gα_s_, e.g. in proximal renal tubules, anterior pituitary, ovary, thyroid gland and the paraventricular nucleus of the hypothalamus [23]–[27]. By contrast, *Gnasxl* is imprinted in the opposite way, and its expression from the paternal allele is limited to a few tissues [15], [18], [28]–[30]. This pattern of transcriptional activity is determined by differential DNA methylation at the main imprinting control region (ICR) at *Nespas* (*Nespas* ICR) and a second differentially methylated region at *exon 1A* upstream of *Gnas*, both of which acquire methylation in the maternal germline and maintain it throughout development in all somatic cells of the offspring (Figure 1) [17], [31]–[33]. The complexity of the locus is further increased by the expression of non-coding RNAs and a third differentially methylated region (termed *Nesp*), which exert epigenetic regulatory functions (Figure 1) [17], [34]–[36].

*Gnas* and *Gnasxl* differ not only in their imprinting, but also in their tissue expression pattern and physiological functions [19], [20]. In contrast to the almost ubiquitous expression of *Gnas*, little is known about the tissue and cell type-specific expression of *Gnasxl*. Limited data indicate an expression pattern mainly restricted to neural, endocrine and some metabolically relevant peripheral tissues, although these were not analysed in histological detail [29], [30], [37]. Furthermore, the expression of *Gnasxl* in neonatal adipose tissue and kidney appears to be silenced towards weaning age, potentially indicating wider developmental changes in its expression pattern [29], [38], [39]. The different and largely opposite physiological functions of *Gnas* and *Gnasxl* have been characterised in knock-out mice, in which the respective first exons were disrupted, and in a mouse model carrying a missense point mutation in exon 6 [23], [25], [30], [39]–[42]. While homozygous deficiency of *Gnas* is embryonically lethal, several tissue-specific homozygous deletions have now been described [20]. However, it is the heterozygous, maternally inherited mutation that displays a distinct imprinting phenotype, due to the maternal allele-specific expression of *Gnas* in some cell types [23], [25], [40]–[42]. These phenotypes include neonatal subcutaneous oedema associated with increased mortality and, in adulthood, resistance to several hormones whose receptors signal via stimulation of cAMP formation (e.g. parathyroid hormone in proximal renal tubules, thyroid stimulating hormone in the thyroid gland) [25], [40], [41]. Mutation of *Gnas* on the maternal allele also causes severe obesity with reduced energy expenditure and type 2 diabetes mellitus-like symptoms (hyperglycemia, glucose intolerance, hyperinsulinemia and insulin resistance) [20], [41], [42]. By analysing brain-specific, maternally inherited *Gnas* deficiency Chen *et al.* showed that this metabolic phenotype could be attributed to a homeostatic function of Gα_s_ in the CNS [23]. A disruption of melanocortin 4 receptor (MC4R) signalling, potentially in the paraventricular hypothalamic nucleus where *Gnas* is imprinted, was implicated as the likely cause for obesity and reduced energy expenditure. These murine phenotypes largely reproduce the symptoms of a human genetic disorder of maternally inherited *GNAS* mutations, termed ‘Albright's Hereditary Osteodystrophy / Pseudohypoparathyroidism type Ia (AHO/PHP-Ia)’ [16], [19].

In comparison, paternally inherited *Gnasxl* mutation causes in many respects an opposite phenotype. At the neonatal stage, *Gnasxl* is required for normal growth, feeding and activity, as deficiency results in leanness, hypoglycemia, general hypotonia and a high mortality rate [14], [30], [40]. This early neonatal phenotype changes towards weaning age, and mutants that survive the first two weeks develop into generally healthy and fertile adults. However, adult *Gnasxl*-deficient mice retain a lean and hypermetabolic phenotype throughout life despite increased food intake, which contrasts with the obesity of maternal *Gnas* mutants [39]. This leanness can be attributed to increased energy expenditure, e.g. adipose tissue lipolysis, which is most likely due to increased activity of the sympathetic nervous system (SNS) and associated with increased glucose tolerance and insulin sensitivity [39]. Apart from the *Gnasxl* knock-out [30], another mouse model, which carries a missense point mutation in exon 6 (the *Oed-Sml* mutation) [40], [42], [43], has been informative in the assessment of phenotypes and how these relate to *Gnasxl* encoded proteins. The exon 6 point mutation renders full-length XLαs (and Gα_s_) non-functional, but does not affect the neural-tissue specific truncated XLN1 protein (Figure 1). Neither does it affect a potential third protein, Alex, which can be translated from a highly unusual second, frame-shifted open reading frame that is contained within the *Gnasxl* first exon [44]. The adult metabolic phenotype of *Sml* mice was found to be largely identical to *Gnasxl* ^m+/p−^ mice, which allowed the conclusion that full-length XLαs is the relevant protein involved in regulation of energy homeostasis [42]. However, with regard to the neonatal feeding phenotype differences were described between both mouse models, which raises the possibility that the XLN1 or Alex proteins have a role at this developmental stage [42]. On a molecular level little is known about their potential functions, since XLN1 lacks the signalling domains encoded by exons 4–12, while Alex was described as a membrane associated protein that interacts with XLαs/XLN1 [44].

To gain insights into the causes of the developmentally changing phenotype of *Gnasxl*-deficient mice, we systematically analysed its expression pattern in the CNS and in peripheral tissues at neonatal and adult stages. In this context we generated a new, conditional *Gnasxl* gene trap mouse model, which expresses a XL-βGalactosidase fusion protein upon Cre recombination, thus providing an excellent histological marker. We also investigated co-localisation of XLαs with neural peptides and enzymes that are involved in the central regulation of energy homeostasis. We identified several developmental changes in the CNS expression pattern, and detected XLαs in areas implicated in the regulation of energy homeostasis and sympathetic outflow. Histological analysis of the hypothalamic arcuate nucleus revealed a reduced activity of the nutrient-sensing kinases mTOR1-S6K, which concurs with the metabolic status and the insulin sensitivity of *Gnasxl* knock-out mice. Surprisingly, in peripheral tissues we determined several types of muscle, including smooth muscle cells of blood vessels, as major sites of expression at the neonatal stage. All muscle expression is silenced in adulthood. The expression in blood vessels accounts for previously obtained *Gnasxl* Northern blot signals from adipose and other peripheral tissues.

## Materials and Methods

### Ethics Statement

All animal work was approved by the Ethical Review Committee of the University of Liverpool and carried out in accordance with MRC guidelines on ‘Responsibility in the use of animals in bioscience research (May 2008)’ and under the authority of the Home Office Project Licence PPL40/3009.

### Mice

Mice (*Mus* musculus) carrying the general *Gnasxl* knock-out mutation (*Gnasxl* ^m+/p−^ after paternal transmission) [30] were maintained on the CD1 outbred genetic background (Charles River, UK). The new conditional gene trap line *XLlacZGT* (strain designation *Gnasxl^tm2Pla^*, according to Targeted Mutation Nomenclature, Jackson Laboratory) was generated in the University of Liverpool Transgenic Unit. Chimaeric *XLlacZGT* founder mice were initially bred to *Flpe*-transgenic mice [45], to remove the frt-flanked *neo^r^*-cassette, and in the following generations *XLlacZGT* females were paired to the CD1 strain background for maintenance of the line and loss of the *Flpe* transgene. Its genetic background is currently mixed 129 / C57BL/6J / CD1. *Nestin-Cre* [46] and *CMV-Cre* [47] transgenic lines were maintained on C57BL/6J strain background. All mice were kept under 12 hrs light/dark cycle with *ad libitum* access to normal chow diet in air conditioned facilities (20–22°C) of the Biomedical Services Unit of the University of Liverpool.

### ES cell culture and Gnasxl conditional gene trap targeting

Mouse embryonic stem (ES) cell culture and G418 selection were carried out as described previously [30], using a male ES cell line (J1) [48], ES-cell certified medium and serum (Thermo Scientific – HyClone), ESGRO® (Millipore) and G418-resistant primary embryonic fibroblasts as feeder cells. The conditional gene trap vector contains the lacZ open reading and polyadenylation signals from pCMVβ (Genbank ID: U02451, Clontech), to which an 80 bp *Gnas* exon 2 splice acceptor sequence (position 190298–190377 bp of Genbank ID: AL593857) was added in frame. The frt-flanked *neomycin* ^r^ cassette was added from pFRT_2_neolacZ [49]. LoxP and lox2272 sites [50] were assembled by oligonucleotide annealing and cloned into the gene trap vector. The sequence of this conditional gene trap plasmid pCLacZGT has been submitted to Genbank ID: JN159854. For conditional gene targeting 5′- and 3′-homologous arms of 5.8 kbp and 2.6 kbp, respectively, were added, resulting in the replacement with targeting cassettes of a 958 bp sequence within intron 1 of *Gnasxl* (position 156559–157516 bp in AL593857), which includes the rarely spliced A20 exon. G418 resistant ES cell colonies were screened by Southern blotting of *Spe*I – digested genomic DNA with a 3.3 kbp *Afl*II fragment external of the homologous arms. One correctly targeted ES cell clone was identified and confirmed in *Mfe*I and *Afl*II genomic DNA digests using *neo* ^r^ and *lacZ* probes, respectively, for Southern blotting. The targeted ES cell clone was injected into C57BL/6J blastocysts, and male chimaeric founder mice were bred to *Flpe* transgenic mice.

### Histology

Tissues from adult mice were collected after perfusion under terminal anaesthesia with 4% PFA/PBS and further fixed for variable times appropriate for the type of histological staining required. For cryostat sections cryo-protection was carried out in 30% sucrose/PBS. XGal staining for XL-βGalactosidase fusion protein activity was carried out on whole-mount tissues or sections as described [51]. Immunohistochemistry was carried out on frozen sections using anti-XLαs antibody (also recognises XLN1; 1∶200 Santa Cruz, sc-18993) with Vectastain Elite Goat IgG kit (Vector Laboratories) and DAB/Ni colour substrate. Double staining of XLαs and Melanin Concentrating Hormone (anti-MCH, 1∶500 Phoenix Pharmaceuticals, H-070-47) was performed as above with post fixing in 4% PFA between XLαs and MCH staining (goat anti rabbit HRP 1∶1000, Jackson ImmunoResearch). Immunofluorescence was carried out on frozen sections. Primary antibodies were used in the following dilutions: 1∶200 for anti-XLαs and anti-pS6 (Cell Signalling Technology, #2211), anti-Tyrosine Hydroxylase (TH) (1∶500, Millipore, AB152), anti-Orexin A (1∶500, Phoenix Pharmaceuticals, H-003-30), anti-α smooth muscle Actin (1∶500, Sigma, A2547), anti-von Willebrand factor (1∶500, Dako, A0082), anti-Choline-acetyltransferase (ChAT) (1∶100, Millipore, AB144P) and anti-βGalactosidase (1∶500, Abcam, ab9361 or 1∶2000, Cappel/MP Biomedicals, #55976). Secondary antibody dilutions were as follows: donkey-anti-goat AF488 (1∶1000, Invitrogen), donkey-anti-chicken DyLight™ 594 (1∶1000, Jackson ImmunoResearch), donkey-anti-rabbit AF594 (1∶1000, Invitrogen) and DAPI (1∶1000, Invitrogen). Percentage co-localisation of Orexin, pS6 and TH with XLαs was assessed by evaluating a representative number of 18 serial coronal brain sections (14 µm), covering the full anterior – posterior extent of the brain region examined from two mice, on an epifluorescent microscope (Zeiss Axioskop 40). pS6 cell number was assessed by evaluating epifluorescent images from 18 serial arcuate nucleus sections per mouse from six pairs of female wild type and *Gnasxl* ^m+/p−^ littermates (age 12 weeks, fed normal chow diet). Co-localisation in blood vessels was examined by confocal imaging (Leica SP2). *In situ* hybridisation for *Corticotropin releasing hormone* (*CRH*) and *Gnasxl* was carried out using Digoxigenin-labelled RNA probes as previously described [30].

### RNA isolation and qRT-PCR

Total RNA was isolated from tissues via homogenisation in Trizol® (Invitrogen). After Trizol/CHCl_3_/Isoamylalcohol extraction a DNA degradation step on gDNA eliminator columns (Qiagen) was included and then an equal volume of 70% EtOH added to the aqueous phase, followed by RNA purification on RNeasy mini columns (Qiagen) according to manufacturer's instructions. The quality of the RNA was assessed by agarose gel electrophoresis, and the concentration determined by absorption at OD_260_. Reverse transcription of 1.75 µg total RNA was carried out using SuperScript III® reverse transcriptase (Invitrogen) and random hexamer primers according to the manufacturer's protocol. For quantitative PCR (qPCR) all samples were reverse transcribed in the same experiment. cDNAs were diluted and qPCR carried out on a Bio-Rad iQ5 cycler using the iQ™ SYBR® Green Supermix (Bio-Rad). qPCR data were normalized to the two housekeeping genes *Gapdh* and *Trf* and evaluated using the ΔΔCt method. PCR primer sequences are provided in Table S1. Standard PCR was carried out using GoTaq® Hot Start Polymerase (Promega).

### Plasma Ghrelin analysis

Adult *Gnasxl* ^m+/p−^ females [30] and their wild-type littermates (aged 5–11 months), which had been fed *ad libitum* with a normal chow diet, were terminally anaesthetized and blood collected by cardiac puncture into heparinised tubes. Samples from all mice were collected during the same circadian time period, between 6–8 hrs into the light phase. Proteinase inhibitor Pefabloc SC (Roche) was added to a final concentration of 1 mg/ml and samples were centrifuged for 15 min, 4°C, 3000 g. Plasma was taken, HCl added to a final concentration of 0.05 M and samples stored at −80°C. Ghrelin was measured using the rat/mouse Ghrelin (active & total) ELISA kit (Linco Research) according to manufacturer's instructions.

### Statistical Analyses

Active Ghrelin plasma data were analysed by non-parametric Mann-Whitney U test; total Ghrelin data were analysed by Students t-test. The number of pS6-positive cells was analysed by non-parametric Wilcoxon's matched-pairs signed-ranks test. The statistics software package Minitab was used.

## Results and Discussion

### A conditional Gnasxl gene trap line provides a lineage marker for expression analysis

**Figure 2:**
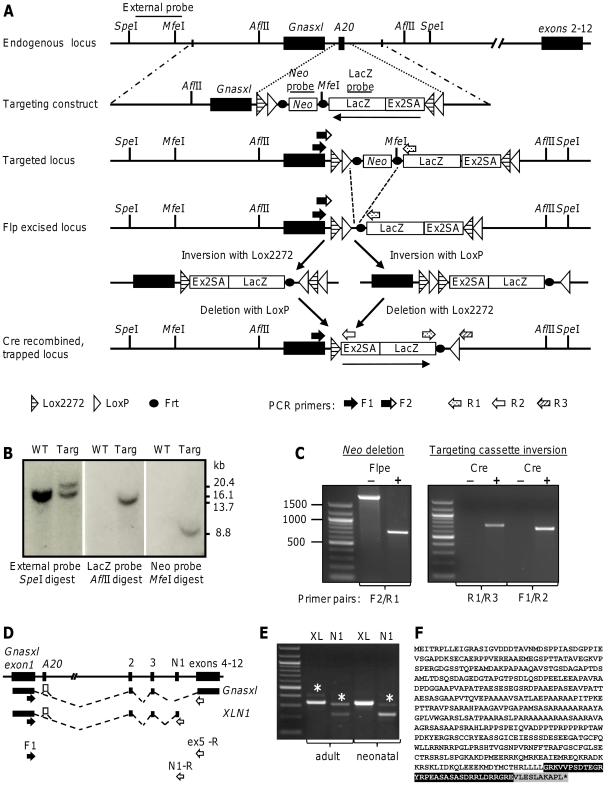
Generation of a conditional gene trap mouse line for (**A**) The targeting construct comprises the *Gnas* exon 2 splice acceptor (Ex2SA) fused in frame to LacZ-pA, an frt-flanked *neo* ^r^ cassette and pairs of loxP/lox2272 sites assembled in head-to-head orientation. The gene trap cassette was inserted in the non-functional (antisense) orientation at the rarely used A20 exon position (see D–F). Cre recombination results in inversion at compatible lox sites followed by excision between head-to-tail sites, which results in a stably integrated gene trap that attracts splicing from *Gnasxl* exon 1. The resulting XL domain – β-Galactosidase (XL-βGal) fusion protein lacks XLαs function, but retains β-Galactosidase activity. Relative positions of probes and restriction sites used in Southern-blots are shown, as are PCR primers (arrows) used in genotyping. (**B**) Southern blots showing a correctly targeted ES-cell clone (*Spe*I: WT = 16.1 kbp, targeted = 20.4 kbp; *Afl*II: targeted = 13.7 kbp; *Mfe*I: targeted = 8.8 kb). (**C**) Genotyping PCRs for deletion of the *neo* ^r^ cassette via *Flpe* mice (left), and for Cre recombinase mediated inversion of the gene trap (right). The Flpe / Cre status of the samples is given above the lanes. For primer locations see (A). (**D**) Scheme indicating splicing of the rarely used A20 exon in full-length *Gnasxl* and neural-specific *XLN1* transcripts. Arrows indicate primers used in (E). (**E**) RT-PCR from wild-type brain using a common *Gnasxl* exon 1 primer combined with reverse primers in exon 5 (full-length *Gnasxl*) or exon N1 (*XLN1*). Exon A20-containing products (size increase: 95 bp) are indicated by asterisks above the respective bands and are hardly detectable in full-length transcripts (XL lanes), but are more prominent in *XLN1* transcripts (N1 lanes). (**F**) Inclusion of the A20 exon results in a frame shift and termination codon in exon 2. The translated *Gnasxl*-A20 sequence from (E) is shown: *Gnasxl* exon 1 sequence (not highlighted), exon A20 sequence (highlighted black), exon 2 sequence (highlighted grey).

To distinguish between functions of *Gnasxl* in development and adulthood and to analyse its roles in different tissues, a conditionally targeted mouse model is required. We employed a gene trap strategy to generate a conditionally targeted allele of *Gnasxl*, since a standard approach of flanking the single *Gnasxl* specific 5′-exon with loxP sites would most likely result in disturbance of the *Nespas* ICR with consequences for the expression of other transcripts of the locus (Figure 1) [33]. The conditional gene trap approach made use of two non-compatible lox site pairs (loxP and lox2272) and Cre recombinase mediated inversion of head-to-head oriented recombination sites, which was followed in this case by excision of intervening sequences and stable activation of the gene trap cassette (Figure 2A) [52]. The gene targeting construct comprised the splice acceptor sequence of *Gnas* exon 2, fused in frame to a LacZ-pA cassette, which served to trap splicing from *Gnasxl* exon 1 and resulted in the expression of an XL domain – βGalactosidase fusion protein (XL-βGal) after Cre recombination. The frt-flanked *neo* ^r^ cassette can be removed by Flp recombinase after ES cell selection [49]. As a location for the gene trap cassette we chose the position of the rarely used A20 exon, which was previously identified in a minority of cDNAs [15]. We confirmed the infrequent inclusion of this exon in *Gnasxl* cDNAs from mouse brain by RT-PCR (Figure 2D, E). Exon A20 containing splice variants were hardly detectable in full-length *Gnasxl* transcripts, but made up a larger proportion of the neural-specific, truncated *XLN1* transcripts. Sequencing of the A20 splice forms revealed an exon size of 95 bp, inclusion of which causes a frameshift and premature termination codon in exon 2 of the *Gnasxl* open reading frame, similar to the human gene (Figure 2F) [15]. Such a truncation of the XLαs protein disrupts its cAMP signalling function, but might be of little relevance for the already truncated XLN1 protein, for which no specific role has been described so far [29]. Therefore, as no specific relevance could be assigned to A20 exon-containing splice variants, we chose to replace it with the gene trap cassettes (Figure 2A). Thus, the gene trap construct removes the exon 2–12 encoded signalling domains from XLαs and also truncates the short XLN1 protein further, but it does not affect the open reading frame of the Alex protein, which is confined to the first exon of *Gnasxl* [44]. During gene targeting one correctly recombined ES cell clone was identified (Figure 2B), which produced several germline transmitting chimaeras. However, transmission of the mutated, inactive gene trap allele from chimaeras (paternal transmission) was associated with an unexpectedly high rate of neonatal mortality among pups, which was reminiscent of general *Gnasxl* ^m+/p−^ knock-out mice [30]. Very few offspring survived in crosses to C57BL/6J; however, survival was improved on a CD1 outbred genetic background. Removal of the frt-flanked *neo* ^r^ cassette through crosses to *Flpe* mice (Figure 2C) [45] did not improve survival, suggesting that *Gnasxl* transcription might have become downregulated by the placement of the gene trap cassette at the A20 exon position. Analysis of *Gnasxl* transcript levels in neonatal brain by qRT-PCR confirmed this hypothesis, indicating a 90% downregulation in paternal mutation carriers, irrespective of whether the gene trap cassette was in its inactive or in its Cre-recombined, active orientation (Figure 2C, Figure S1). Further investigations are currently under way to determine the cause of the unexpected downregulation in *Gnasxl* expression levels. Although this reduction of *Gnasxl* expression levels compromised phenotype analysis of conditional, Cre-induced gene trap mutants, the activated gene trap generated a readily detectable XL-βGal fusion protein. This provided a highly useful and faithful marker for the histological analysis of the *Gnasxl* expression pattern and its developmental changes in central and peripheral tissues.

### Developmental changes in the brain expression pattern of Gnasxl correlate with phenotype changes of knock-out mice

**Figure 3:**
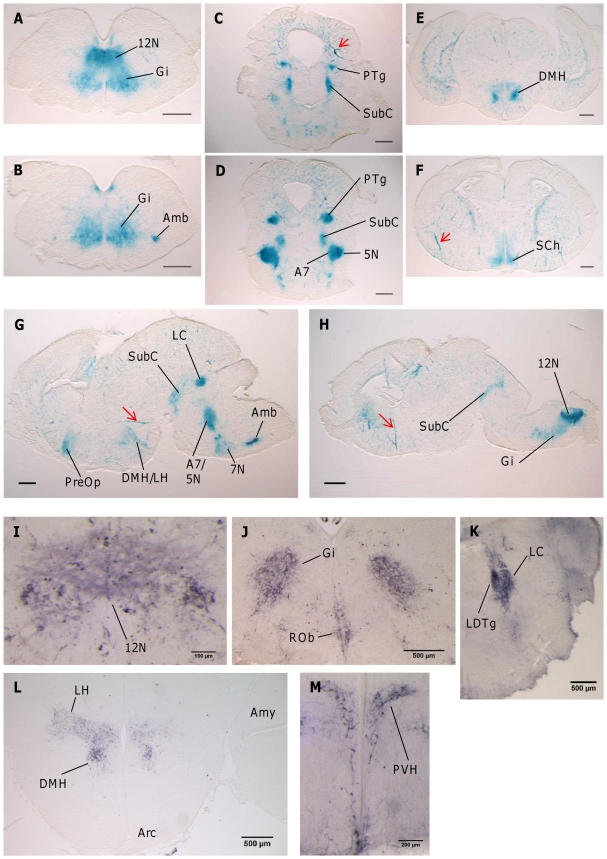
Expression of *Gnasxl* in neonatal brain (P1). (**A–H**) XGal staining of neonatal brain sections (A–F coronal sections; G, H sagittal sections), indicating XL-βGal fusion protein activity in *Nestin-Cre*/+; +/*XLlacZGT* offspring. Images are shown in posterior to anterior order covering the medulla oblongata (A, B), pons (C, D) and hypothalamus (E, F). Sagittal sections are located peripherally (G) and close to the midline (H). Red arrows indicate expression in blood vessels (compare Figure 7A–G). (**I–M**) Immunohistochemistry for XLαs on coronal sections of the medulla oblongata (I, J), pons (K) and hypothalamus (L, M) of wild-type brain. 5N – motor trigeminal nucleus, 7N – facial nucleus, 12N – hypoglossal nucleus, A7 – A7 noradrenaline cells, Amb – ambiguus nucleus, Amy – medial amygdaloid nucleus, Arc – arcuate hypothalamic nucleus, DMH – dorsomedial hypothalamic nucleus, Gi – gigantocellular reticular nucleus, LC – locus coeruleus, LDTg – laterodorsal tegmental nucleus, LH – lateral hypothalamic area, PreOp – preoptic area, PTg – pedunculopontine reticular nucleus, PVH – paraventricular hypothalamic nucleus, ROb – raphe obscurus nucleus, SCh – suprachiasmatic nucleus, SubC – subcoeruleus nucleus. Scale bars = 500 µm or as indicated.

To analyse the functionality of the gene trap construct we crossed male *Gnasxl* gene trap carriers (*XLlacZGT*) with *Nestin-Cre* transgenic females [46] and assessed the expression of the XL-βGal fusion protein by XGal staining of neonatal brain sections of double positive offspring. Fusion protein activity was readily detected in novel, as well as previously identified, mid- and hindbrain regions (Figure 3A–H), thus confirming the correct spacial transcriptional activity of the *Gnasxl* gene trap [30]. These regions comprised the orofacial motor nuclei (hypoglossal (12N), facial (7N) and motor trigeminal nuclei (5N)), the noradrenergic locus coeruleus (LC) as well as the gigantocellular reticular area (Gi) and the nucleus ambiguus (Amb) of the medulla (Figure 3A, B, D, G, H). Additional novel sites of expression included the anterior pons, i.e. the pedunculopontine tegmental nucleus (PTg) and the subcoeruleus area, including the noradrenergic A7 nucleus (Figure 3C, D). *Gnasxl* expression was also discovered in several regions of the neonatal hypothalamus, including the dorsomedial (DMH) and lateral areas (LH), the suprachiasmatic nucleus (SCh) and preoptic area (Figure 3E–G). No XGal staining was observed in control brain tissues (Figure S2A). Immunohistochemical analysis using an XLαs-specific antibody (which also recognises the truncated neural variant XLN1) on wild-type brain sections was consistent with the gene trap expression pattern and *in situ* hybridisation data [30], as it showed expression in the 12N, Gi and raphe obscurus (ROb) of the medulla as well as the LC and laterodorsal tegmental nucleus (LDTg) of the pons (Figure 3I–K). The LDTg and the PTg constitute major cholinergic centres of the brain [53]. In the hypothalamus the paraventricular nucleus (PVH) was identified as an XLαs-positive area in addition to the DMH and LH (Figure 3L, M). Neonatal PVH expression was consequently confirmed in XGal stained coronal sections of gene trap tissue (data not shown). The specificity of the XLαs antibody was confirmed using brain sections from *Gnasxl* ^m+/p−^ mice, which did not result in any staining (Figure S2C, D). In the arcuate nucleus (Arc) of the hypothalamus no protein was detected on postnatal day 1, but *Gnasxl* transcripts were expressed by day 4 (Figure S3A).

This pattern of expression in defined subregions of the brainstem and midbrain, which includes nuclei involved in the control of the autonomous nervous system, is highly suggestive in the context of the neonatal phenotype of *Gnasxl*-deficient mice [30]. As the mouse brain continues to develop during the first postnatal week, *Gnasxl* expression is found to be strongest in brainstem nuclei that have to be functional soon after birth, e.g. in the motor nuclei that innervate orofacial muscles (5N, 7N, 12N) [54]–[56]. The impaired feeding of *Gnasxl*-deficient pups [14], [30] correlates with the expression in these nuclei and with a role of cAMP signalling in orofacial motoneuron activity [55]. However, the function of XLαs in these neurons remains to be determined, and additional causes for this phenotype cannot be excluded (see below). Similarly, the expression of XLαs in other brainstem nuclei is likely to be relevant for the relative inactivity seen in *Gnasxl*-deficient neonates as, for example, the LC, LDTg, PTg and Gi influence arousal states, wakefulness and sleep through their widespread projections within the brain [57]–[60]. Additionally, the ROb is implicated in SNS and autonomic motor outflow [61], and the Amb constitutes a major centre of vagal, parasympathetic outflow to the heart [62], suggesting that XLαs might impact on heart rate in mice. By contrast, the hypothalamus and its neural circuits are still under development during the first postnatal week [63], [64], which makes an essential neonatal function of XLαs in this brain region less likely. However, it cannot be excluded that XLαs deficiency impacts on hypothalamic development and results in lasting effects on adult energy homeostasis regulation, as has been shown in the case of leptin deficiency [63].

**Figure 4:**
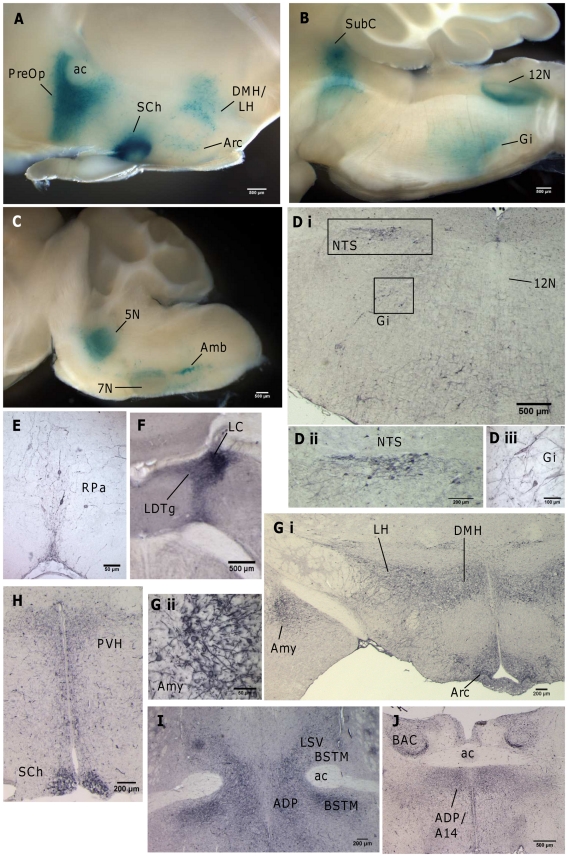
Expression of *Gnasxl* in the adult brain. (**A–C**) XGal staining of *CMV-Cre*/+; +/*XLlacZGT* sagittal brain vibratome section (thickness: 500 µm) indicating XL-βGal fusion protein activity in the hypothalamus (A) and medulla ((B) midsagittal, (C) parasagittal). Note the reduced expression levels in the 7N compared to the neonatal stage (compare Figure 3G and [30]). (**D–J**) Immunohistochemistry for XLαs on coronal wild-type brain sections in caudal (medulla) to rostral (preoptic area) order. Changes from the neonatal pattern include downregulation of expression in the 12N (D i), silencing in the LDTg of the pons (F), novel expression in the nucleus tractus solitarius (D) and medial amygdala (G), and more widespread expression in several hypothalamic nuclei (G, H) and the preoptic area (I, J). Note that in (F) the darkly stained area indicates the neuronal cell bodies of the LC, while the surrounding lighter stained area represents the dense network of neurites emerging from the LC. Higher magnification images of the region show no XLαs signals in the neurons of the LDTg. 5N – motor trigeminal nucleus, 7N – facial nucleus, 12N – hypoglossal nucleus, A14 – A14 dopamine cells, ac – anterior commissure, ADP – anterodorsal preoptic nucleus, Amb – ambiguus nucleus, Amy – medial amygdaloid nucleus, Arc – arcuate nucleus, BAC – bed nucleus of the anterior commissure, BSTM – bed nucleus of the stria terminalis (medial parts), DMH – dorsomedial hypothalamic nucleus, Gi – gigantocellular reticular nucleus, LC – locus coeruleus, LDTg – laterodorsal tegmental nucleus, LH – lateral hypothalamic area, LSV – lateral septal nucleus (ventral part), NTS – nucleus tractus solitarius, PreOp – preoptic area, PVH – paraventricular hypothalamic nucleus, RPa – raphe pallidus, SCh – suprachiamatic nucleus, SubC – subcoeruleus nucleus.

As the phenotype of *Gnasxl*-deficient mice changes from an often lethal failure-to-thrive in the first postnatal week to an overall healthy, but hypermetabolic and lean, physiology in surviving adults [30], [39], we analysed whether this might be based on changes in the expression pattern of the gene. To obtain an initial overview, we undertook XGal staining of vibratome sections of adult brain from mice carrying the Cre-activated gene trap. These indicated some areas of continued expression of *Gnasxl*, e.g. in the Amb, Gi, 12N, 7N and 5N of the medulla, the LC and SubC of the pons and the DMH, LH and SCh of the hypothalamus (Figure 4A–C). Although the three orofacial motor nuclei stained for the XL-βGal fusion protein (Figure 4B, C), we were unable to detect XLαs protein in these areas in tissue sections from adult wild-types by immunohistochemistry (Figure 4D), and only obtained weak staining of a small number of neurons by *in situ* hybridisation (data not shown). These results indicate low, downregulated expression levels in the motor nuclei that, however, still produce a blue XGal precipitate in overnight stained 500 µm thick vibratome sections. A downregulation and change in requirement for XLαs in orofacial motor nuclei would concur with the gain of normal feeding capabilities at weaning and increased food intake of adult *Gnasxl* knock-out mice [39]. To further investigate the adult food intake phenotype, we analysed the plasma levels of the peptide hormone Ghrelin, which is produced by the stomach and stimulates food intake via actions in the hypothalamus and brainstem [65]. In line with the increased food intake data, we found total and active (acylated) Ghrelin levels to be elevated in adult *Gnasxl* ^m+/p−^ mice (total Ghrelin: *Gnasxl* ^m+/p−^ = 7.53±0.87 ng/ml, WT = 3.19±0.31 ng/ml, p<0.0001; active Ghrelin: *Gnasxl* ^m+/p−^ = 443.86±77.85 pg/ml; WT = 200.74±27.82 pg/ml, p = 0.003; means ± SEM; n = 13–15; Figure S4). These data support the view that the food intake promoting functions of Ghrelin are not impaired by lack of XLαs. Furthermore, it appears likely that the elevated Ghrelin and food intake levels of *Gnasxl*-deficient mice constitute a compensatory physiological mechanism to counterbalance their hypermetabolic phenotype.

**Figure 5:**
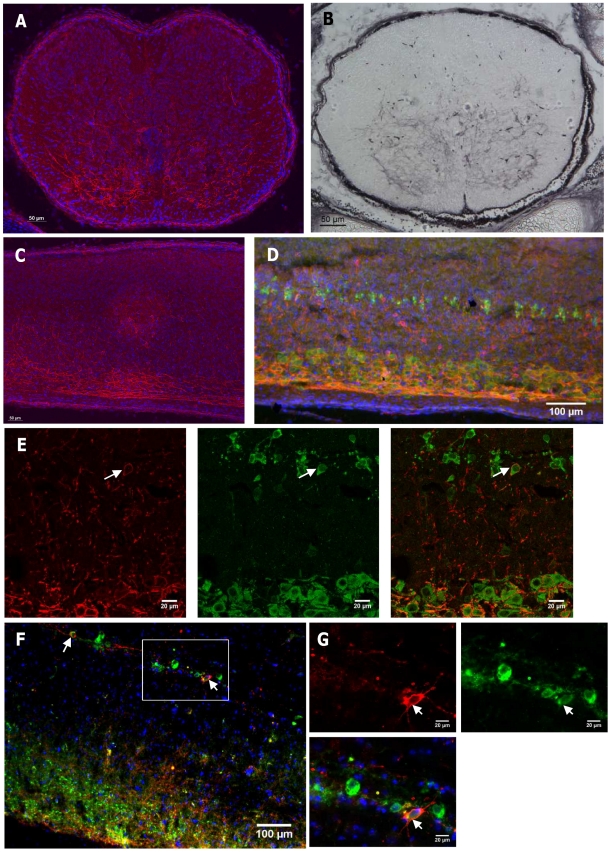
Spinal cord expression of *Gnasxl*. (**A–C**) Immunohistochemistry of neonatal (P1) thoracic spinal cords from *Nestin-Cre/+*; +/*XLlacZGT* mice using an anti-βGalactosidase antibody (red, A and C), or from wild-type mice using an anti-XLαs antibody (purple DAB/Ni staining, B). Transverse (A, B) and sagittal (C) sections are shown. *Gnasxl* expression is detected in scattered neurons of the intermediolateral region as well as in the ventrolateral, motoneuron containing area. (**D–G**) Co-staining for XL-βGal fusion protein (red) and Choline acetyltransferase (ChAT; green) on sagittal sections from neonatal (D, E) and adult (F, G) spinal cords. ChAT marks cholinergic sympathetic preganglionic neurons of the intermediolateral layer as well as ventrolateral motoneurons. While XLαs and ChAT are co-expressed in the majority of motoneurons, they are only occasionally co-localised in neurons of the intermediolateral layer (white arrows in (E–G)). Epifluorescent (D, F, G) and confocal (E) images are shown. (G) Shows a magnification of the area indicated in (F). Note the plasma membrane association of XL-βGal fusion protein, due to palmitoylation of the XL domain [97]. Neonatal tissues in (D, E) were obtained from *Nestin-Cre*/+; +/*XLlacZGT* offspring, while adult samples in (F, G) were derived from *CMV-Cre*/+; +/*XLlacZGT* mice.

Further histological analysis of XLαs also identified novel, adult-specific expression in the nucleus tractus solitarius (NTS) (Figure 4D), a brainstem area involved in regulation of energy homeostasis [66]. In the ventral medulla expression was found in the raphe pallidus (RPa) (Figure 4E), which, like the ROb, constitutes part of the sympathetic outflow chain, e.g. towards adipose tissue [61], [67], [68]. The RPa already stained XLαs-positive at neonatal stages [30] (and data not shown). Another noticeable change of *Gnasxl* expression was observed in the cholinergic LDTg of the pons, which was silenced at the adult stage, while the neighbouring noradrenergic LC retained expression (Figure 4F). XLαs expression in the four hypothalamic nuclei (Arc, DMH, LH, PVH) appeared more widespread in adult as compared to neonatal tissue (Figure 4G, H), and high levels of expression were found in the SCh at both stages (Figure 4A, H and 3F, Figure S3A). Adult-specific XLαs staining was identified in a medial part of the amygdala close to the optic tract (Figure 4G). High levels of expression, which developed from early postnatal stages onwards, were also apparent in the preoptic area ventral and rostral of the anterior commissure (Figure 4A, I, J). Immunohistochemistry identified several subregions of the bed nucleus of the stria terminalis, the anterodorsal preoptic nucleus, the A14 dopaminergic area and parts of the lateral septal nucleus as XLαs-positive (Figure 4I, J). As subregions of the preoptic area have been implicated, among other functions, in the control of body temperature and sleep / arousal states [69]–[71], it is notable that XLαs expression also correlates on this CNS level with areas of autonomic and homeostatic control. Furthermore, to investigate another part of the hierarchical organisation of autonomic systems that might be relevant for the increased SNS activity of XLαs deficient mice [39] (and our own unpublished data), we investigated whether expression occurs in the spinal cord, which contains sympathetic preganglionic neurons (SPNs) [68], [69]. We detected expression of XLαs in scattered neurons of the intermediolateral region of the spinal cord as well as in ventrolateral, motoneuron-containing areas at neonatal and adult stages (Figure 5A–C). To further analyse whether XLαs expression occurs in SPNs, we determined its co-localisation with Choline acetyltransferase (ChAT), which serves as a marker for SPNs as well as ventral motoneurons. We found co-expression of the XL-βGal fusion protein in a large proportion of cholinergic motoneurons. In the intermediolateral layer the two proteins were mostly located in separate populations, with few neurons showing co-expression. This was the case for neonatal and adult stages (Figure 5D–G). Our findings indicate that XLαs is less likely to exert a crucial SNS-related function directly in SPNs. However, XLαs-positive cells and neurites were often found in the vicinity of SPNs, suggesting that they might have a regulatory influence on the latter. A summary of the developmentally changing CNS expression pattern of *Gnasxl* is presented in Table S2.

One of the main findings of this expression analysis is the association of XLαs with brain regions involved in the regulation of SNS outflow. Multisynaptic retrograde tracer experiments have identified the hierarchical organisation of SNS control centres, which include the SPNs of the intermediolateral spinal cord, several regions of the medulla oblongata (RPa, ROb, Gi, NTS), as well as specific midbrain and hypothalamic regions (PVH, DMH, LH, Arc, PreOp) [61], [68], [69]. Recent work has especially emphasised the roles of the preoptic area and the DMH, both of which express XLαs extensively, in controlling the medullary raphe and SNS-mediated thermoregulation [70]–[72]. Furthermore, the melanocortin/MC4R neuropeptide signalling system localises to several of these sympathetic control regions and stimulates energy expenditure, brown adipose tissue (BAT) thermogenesis, sympathetic nerve activity and heart rate [67], [73]–[76]. Since *Gnasxl*-deficient mice show elevated energy expenditure, sympathetic outflow and cardiovascular parameters [39] (and our own unpublished data), it appears likely that a hyperactive melanocortin system could, at least partly, account for the knock-out phenotype. This hypothesis is supported by the finding that the MC4R signals via Gα_s_ *in vivo*, as was shown by a brain-specific knock-out of the maternal *Gnas* allele, which partially recapitulates the obese, hypometabolic, bradycardic and melanocortin-resistant phenotype of the *Mc4r* knock-out [23], [77], [78]. Therefore, the largely opposite energy homeostasis phenotypes of the *Gnas* and *Gnasxl* knock-out mice suggest that XLαs might act to antagonise the melanocortin pathway and suppress SNS outflow [23], [39], [41]. However, the *in vivo* mechanisms of XLαs function and receptor coupling remain to be determined.

### Co-expression analyses of XLαs with hypothalamic neuropeptides and reduced activity of the mTOR1–S6K signalling pathway, which regulates insulin and leptin sensitivity

**Figure 6:**
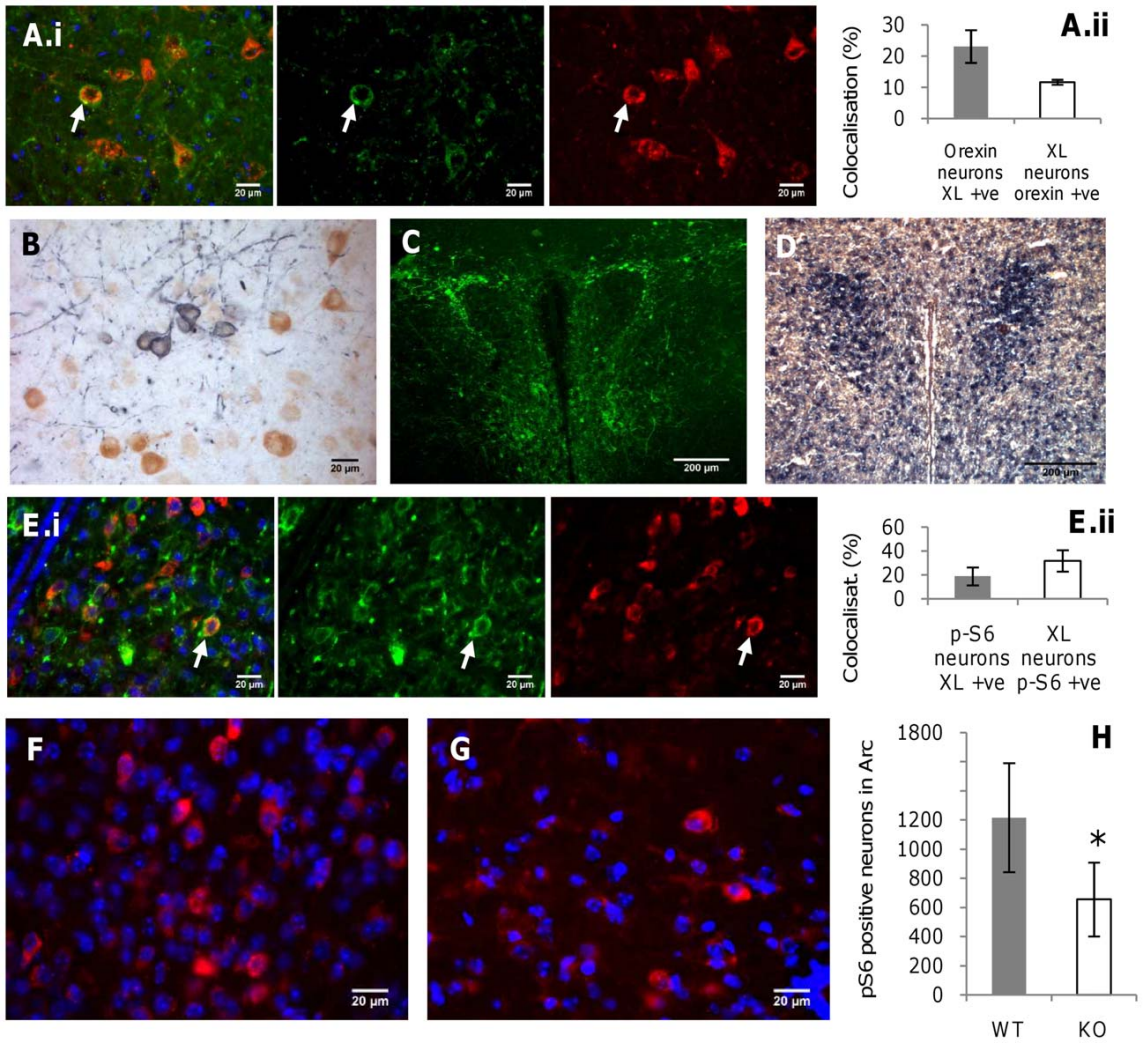
XLαs co-localisation with hypothalamic neuropeptides and assessment of arcuate nucleus mTOR1-S6K activity in *Gnasxl* ^m+/p−^ mice. (**A**) A representative image of co-localisation of XLαs (green) with Orexin A (red) in the DMH/LH is shown (i) and quantification of co-localised cell body staining is provided on the right (ii). (**B**) XLαs (purple) and MCH (brown) are expressed in separate neuron populations in the DMH/LH. (**C, D**) Immunofluorescence staining for XLαs (C) and *in situ* hybridisation for CRH (D) in neighbouring hypothalamic sections indicate separate expression domains in peripheral and central subdivisions of the PVH, respectively. (**E**) Co-localisation of pS6, a substrate and marker of mTOR1-S6K activity, in XLαs-positive neurons of wild-type Arc tissue. A representative image (i) and quantification (ii) of a series of sections from two mice (age 12 weeks) is shown. ∼32% of XLαs-positive neurons show pS6 signals under *ad libitum* normal chow fed conditions. (**F–H**) Reduced number of pS6 positive neurons in the Arc of *Gnasxl* ^m+/p−^ mice (aged 12 weeks on normal chow diet). Representative images of pS6 immunofluorescence of wild-type (F) and *Gnasxl* ^m+/p−^ (G) Arc sections are shown, and a quantification is provided in (H), (n = 6; p = 0.036). See materials and methods for details of the quantification method.

Since hypothalamic neuron populations and signalling pathways that regulate food intake and energy expenditure have been well characterised over recent years [65], [79], [80], we investigated potential co-localisation of XLαs with relevant neural markers. In the DMH/LH two neuron populations, expressing Orexin and MCH, have diverse effects on energy balance. Although both stimulate food intake, Orexin also stimulates energy expenditure, while MCH has the opposite effect [65], [79], [81]. We found XLαs to be expressed in a proportion of Orexin neurons (∼23%), but no co-localisation was detected in MCH neurons (Figure 6A, B). An increased activity of the Orexin pathway in *Gnasxl* knock-out mice could contribute to the elevated food intake and energy expenditure observed [39], [81]. In the PVH, XLαs was detected in peripheral areas, but was not expressed in the central subdivisions, which contain CRH neurons, a neuropeptide that stimulates SNS outflow and energy expenditure (Figure 6C, D) [82]. Further studies, with a marker for catecholaminergic neurons (TH), showed co-expression not only in the noradrenergic neurons of the locus coeruleus and the subcoeruleus area of the pons (data not shown), but also in the A12 dopaminergic neurons of the Arc (65% of TH neurons positive for XLαs; Figure S3B), which regulate prolactin secretion from the pituitary gland [83]. By contrast, no co-expression was found in the A13 dopaminergic cells of the zona incerta (Figure S3Bi). Similarly, no XLαs expression was detectable in the dopaminergic neurons of the ventral tegmental area and substantia nigra (data not shown).

Neurons of the hypothalamic Arc influence energy homeostasis in response to circulating levels of the peptide hormones leptin and insulin [65], [79], [80]. Furthermore, we have previously shown that XLαs-deficient mice are hypoglycemic, have significantly lower plasma levels of both hormones and show peripheral insulin sensitivity [39]. One of the intracellular regulators of leptin and insulin receptor signalling is the mTOR1–S6K pathway, which constitutes a key integrator of cellular nutrient sensing and metabolism in peripheral tissues as well as the hypothalamus. mTOR1-S6K are activated by various stimuli, including signal transduction from insulin and leptin receptors via IRS1–PI3K–Akt (Figure S5) [80], [84]. In a negative feedback loop mTOR1-S6K mediate phosphorylation of IRS1, thus desensitising and inhibiting the PI3K–Akt signalling branch of the insulin and leptin receptors, which can lead to hormone resistance. It has also been shown that metabolically relevant mTOR1-S6K signalling specifically occurs in Arc NPY/Agrp and POMC neurons [85]. To investigate potential differences in this pathway in Arc neurons of XLαs-deficient mice, we first analysed co-localisation of XLαs with pS6, a substrate and marker of S6K activity, in wild-type tissue sections. Next, we assessed pS6 signals (number of pS6 positive neurons) comparatively in the Arc of WT versus *Gnasxl* ^m+/p−^ mice, to test whether the metabolic status of the knock-out mice correlates with a reduced activity of mTOR1-S6K. In WT mice under *ad libitum* chow fed conditions ∼32% of XLαs-expressing Arc neurons showed a pS6 signal (Figure 6E). Under the same standard feeding conditions the number of pS6 positive neurons in the Arc of *Gnasxl* ^m+/p−^ mice was significantly lower than in WT littermates (reduced by 46%; n = 6; p = 0.036) (Figure 6F–H). These data confirm our hypothesis of reduced activity of the mTOR1-S6K kinase pathway in Arc neurons of XLαs deficient mice, which concurs with their metabolic status. Insulin injection in *Gnasxl* ^m+/p−^ mice results in a stronger activation of the IRS1-PI3K-Akt pathway in peripheral tissues when compared to WT controls, indicating a generally increased insulin sensitivity [39]. CNS sensitivity to insulin and/or leptin of *Gnasxl*-deficient mice will be explored in future experiments. It is interesting to note that S6K-deficient mice exhibit a similar metabolic phenotype as adult *Gnasxl* ^m+/p−^ mice, including reduced body weight and adiposity, and increased food intake, lipolysis, energy expenditure and insulin sensitivity [39], [86]. It currently remains uncertain whether the reduced activity of the mTOR1-S6K pathway is involved in causing the lean and hypermetabolic *Gnasxl* ^m+/p−^ phenotype, or whether this is a consequence of a metabolic condition that originates from other factors.

### Peripheral Gnasxl expression is mainly limited to muscle tissues, including smooth muscle cells of blood vessels, and is silenced at adult stages

**Figure 7:**
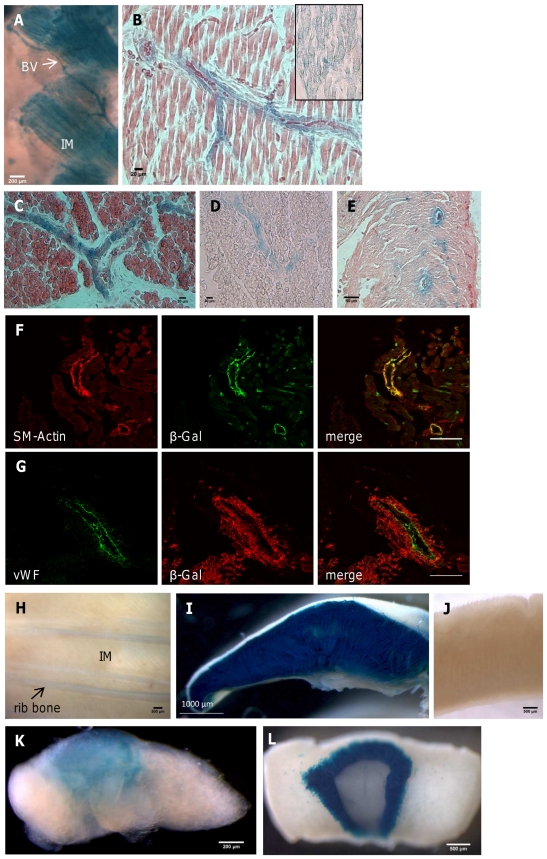
Expression of *Gnasxl* in peripheral tissues of *Cre*/+; +/*XLlacZGT* mice. Either the *Nestin-Cre* (A, B) or the *CMV-Cre* (C–L) transgenic line was used to activate the gene trap cassette. (**A**) XGal whole-mount staining of neonatal (P1) intercostal muscle and blood vessels. (**B**) XGal, eosin counter-stained section of P1 skeletal muscle from hind limb showing expression of the XL-βGal fusion protein in muscle fibres and blood vessels (inset without eosin counter-staining depicts muscle fibre staining for clarity). (**C, D**) Blood vessel XGal staining in neonatal brown adipose tissue with (C) and without (D) eosin counter-stain. (**E**) XL-βGal fusion protein activity in blood vessels of the heart, P1. (**F, G**) *Gnasxl* is expressed in smooth muscle cells, but not endothelial cells, of blood vessels. Confocal imaging of neonatal blood vessels using antibodies for βGalactosidase and either α-smooth muscle Actin (F) or von Willebrand factor (G). (**H**) Expression of XL-βGal fusion protein in intercostal muscle and blood vessels is silenced at the adult stage. XGal staining of adult whole-mount tissue in comparison to (A). (**I, J**) XL-βGal expression in neonatal tongue muscle (I) is also silenced at the adult stage (J); XGal whole-mount staining of longitudinally sectioned tissue samples. (**K, L**) Expression of *Gnasxl* in the intermediate lobe of the neonatal pituitary (K) is maintained at the adult stage (L). XGal staining of whole-mount tissues. BV = blood vessel, IM = intercostal muscle. For controls see Figure S2.

Our previous data on *Gnasxl* expression in peripheral neonatal tissues, which was limited to Northern blot analysis, indicated expression in brown and white adipose tissue, heart, kidney and gastrointestinal tissues [30]. To investigate these findings on a histological level, we analysed expression of the XL-βGal fusion protein in crosses of male *XLlacZGT* carriers to *Nestin-Cre* and *CMV-Cre* females [46], [47]. In *Nestin-Cre*/+; +/*XLlacZGT* mice, we initially noticed in whole-mount XGal stainings of neonatal spine preparations that not only neural tissue of the spinal cord stained positive, but also surrounding intercostal muscle. A more detailed analysis confirmed expression in intercostal muscle and additionally revealed expression in blood vessels passing through the tissue (Figure 7A). No staining was observed in control muscle samples from wild-type or single transgenic Cre or gene trap littermates (Figure S2B). Further examination of skeletal muscle of the hind limbs also showed expression of the XL-βGal fusion protein in muscle fibers and more strongly in blood vessels (Figure 7B). This was confirmed in wild-type tissue sections using XLαs immunohistochemistry (Figure S2E, F). Although the expression of the *Nestin-Cre* transgene is widely regarded as nervous system-specific [46], our results agree with a previous report, which detected Cre activity outside the nervous system, e.g. in the somite – myotome lineage during embryonic development [51]. Thus, the Cre activity of this transgenic line does reflect the wider expression pattern of the *Nestin* gene, which includes muscle precursors [87]. The finding of *Gnasxl* gene trap activity in blood vessels raised the possibility that previously obtained Northern blot signals from various other tissues might in fact be due to blood vessel contamination. Histological examination of neonatal brown adipose tissue and heart confirmed this hypothesis as XL-βGal fusion protein activity was only detected in blood vessels and not, for example, in mature adipocytes (Figure 7C–E). Similar blood vessel staining was also found in neonatal white adipose tissue and pancreas (data not shown). To determine the cell type within blood vessels that expresses *Gnasxl*, we examined co-localisation of XL-βGal with markers for smooth muscle and endothelial cells (α-smooth muscle Actin and von Willebrand factor, respectively) using confocal microscopy. XL-βGal expression was detected in the smooth muscle cells, but not endothelial cells, of blood vessels (Figure 7F, G). Similar to skeletal muscle, vascular smooth muscle cells are partly derived from the embryonic mesoderm / somite lineage [88] and, therefore, *Gnasxl* expression is likely to reflect the common embryonic origin of these tissues. Following reports that *Gnasxl* mRNA and protein are downregulated in adipose tissue and kidney from mid-postnatal stages onwards [38], [39], we examined expression of the XL-βGal fusion protein in adult tissues. No βGalactosidase activity could be detected in whole mount XGal stainings of adult intercostal muscle preparations, which also contained blood vessels (Figure 7H). Skeletal muscle preparations from the hind leg were negative for XL-βGal activity, too (data not shown). Another muscle tissue, the tongue, followed a similar pattern, as strong expression was found at neonatal stages, while expression was silenced in the adult tissue (Figure 7I, J). The expression in neonatal tongue muscle was confirmed using XLαs immunohistochemistry on wild-type tissue sections (Figure S2G, H). Blood vessels of other adult tissues were also negative for XL-βGal expression, e.g. in brain (compare Figure 3C, F, G, H with Figure 4A–C) and adipose tissue (data not shown).

Additionally to the characterisation of these novel peripheral expression sites, we also examined the pituitary and adrenal glands, since they had previously been shown by *in situ* hybridisation to express *Gnasxl* mRNA [30]. In line with the earlier data the neonatal pituitary stained positive for XL-βGal activity in the intermediate lobe, which further confirms the correct functionality of the conditional gene trap cassette (Figure 7K; control in Figure S2I). However, in contrast to muscle tissue, *Gnasxl* expression remained in the adult pituitary (Figure 7L) [29]. The melanotroph cells of the intermediate lobe produce the *Pomc* gene-derived peptide αMSH, but little is known about their physiological function. Another neuroendocrine tissue, the adrenal medulla, also showed XL-βGal fusion protein activity at neonatal and adult stages (Figure S6). As part of the sympathetic stress response the adrenal medulla secretes mainly epinephrine, which has been shown to be elevated in *Gnasxl* ^m+/p−^ mice [39] and might contribute to their hypermetabolic phenotype. The histological analysis of *Gnasxl* expression in kidney and gastrointestinal tissues [30] was complicated by endogenous βGalactosidase-like enzyme activity in control samples (Figure S2J).

In summary, we have now associated the characteristic phenotypes of neonatal and adult *Gnasxl*-deficient mice [30], [39] with the changing expression pattern of the gene. One characteristic feature of mutant pups is their reduced suckling activity, which was previously attributed to a potential role of XLαs in orofacial motor nuclei [30]. Our data now show that the neonatal expression of XLαs in these motor nuclei is downregulated, but not completely silenced, at adult stages. In correlation with this change in expression, food intake is no longer impaired in adult *Gnasxl* ^m+/p−^ mice [39]. However, the newly identified, transient high levels of XLαs expression in neonatal muscle tissues, especially tongue muscle, raise the possibility that the feeding difficulties of mutants might be caused by a dysfunction of this muscle tissue itself. XLαs might also have a role in skeletal muscle at the neonatal stage, which could explain the relative inactivity and inertia observed in deficient pups [14], [30]. Muscle-specific disruption of *Gnasxl*, using a modified version of this gene trap line (currently in progress), will be required to validate this hypothesis. Such an experiment would also clarify whether the truncated neural-specific XLN1 protein (Figure 1) has a role in neonatal feeding, as has been suggested by Kelly *et al.* [42]. Mice with a paternally inherited exon 6 mutation (*Sml*), which disrupts full-length XLαs but not XLN1, showed normal suckling activity. Comparing their findings to *Gnasxl* ^m+/p−^ mice, in which all three proteins (XLαs, XLN1 and Alex) are deleted, the authors conclude that XLN1 might have a role in neonatal feeding [30], [42]. Although such a function of XLN1 cannot be excluded at this stage, other factors can also contribute to differences in feeding efficiency of *Sml* and *Gnasxl* ^m+/p−^ pups. It is well documented that differences in genetic background, on which the two strains are kept, influence postnatal survival; neither *Oed-Sml* nor *Gnasxl* ^m+/p−^ are viable on some strain backgrounds [30], [42]. Overall, *Sml* and *Gnasxl* ^m+/p−^ pups show very similar postnatal body weight trajectories [30], [40].

Since the expression of XLαs in peripheral tissues is widely silenced towards adulthood, it is highly likely that the lean and hypermetabolic phenotype of *Gnasxl* ^m+/p−^ mice is due to a lack of function in the CNS. Overall, the adult CNS pattern of XLαs expression correlates remarkably well with the hierarchical organisation and control of sympathetic outflow on several levels, including the preoptic area, Arc, DMH/LH, PVH, medullary raphe, NTS and spinal cord [61], [69], [72], [75], [76]. Our finding that XLαs is located in a neuron population of the intermediolateral spinal cord that is mostly different from, but often in the vicinity of, the cholinergic SPNs suggests that it might play a role in a pathway parallel to the SNS, i.e. in neurons that exert an inhibitory influence on SNS outflow. Such an antagonistic influence on the SNS could occur on the level of the spinal cord as well as the medulla or hypothalamus. Little is known about pathways that utilise cAMP stimulating receptors to antagonise or inhibit metabolic rate and SNS outflow, i.e. pathways in which XLαs could act directly. By contrast, several stimulatory G-protein coupled receptors are known to increase energy expenditure and SNS functions, for example the melanocortin receptor MC4R and the Glucagon-like peptide 1 receptor (GLP-1R) [74], [77], [89].

The regulation of postnatal growth and adult energy homeostasis has emerged as a common theme, on which, apart from the *Gnas* locus with its two major protein products Gα_s_ and XLαs, a number of imprinted genes exert influence [6], [9]. In fact, it was recently shown that the imprinted adaptor protein Grb10 is a direct target of mTOR1 and inhibits insulin / insulin-like growth factor receptor signalling [90], [91]. Accordingly, mice lacking Grb10 expression from the maternal allele show a phenotype that is characterised by overgrowth and increased insulin sensitivity [92]. Furthermore, like *Gnasxl*, several imprinted genes have roles in the CNS [10], [12]. The Prader-Willi and Angelman Syndrome (PWS-AS) locus, for example, contains a cluster of imprinted genes with neural functions. *Magel 2* and *Necdin* are involved in the central stimulation of neonatal suckling and respiration, respectively [93], [94]. In adult mice *Magel 2* affects Orexin expression and feeding behaviour and additionally regulates circadian rhythmicity in the suprachiasmatic nucleus [11], which also expresses high levels of *Gnasxl*. The maternally expressed Angelman Syndrome protein Ube3A, on the other hand, is required for synapse formation and synaptic plasticity [95]. These findings and the recent discovery of a large number of monoallelically expressed transcripts in the brain [96] demonstrate that the CNS constitutes an important tissue, through which imprinted genes influence postnatal development and adult homeostatic functions.

## Supporting Information

Figure S1 **Analysis of** ***Gnasxl*** **transcript levels by qRT-PCR.** RNA from neonatal brain of wild-type, *Cre*/+; +/*XLlacZGT* (inverted, active gene trap) and +/+; +/*XLlacZGT* (inactive gene trap) littermates was analysed using primers specific for *Gnasxl* exon 1. Expression levels were normalised to the housekeeping genes *Gapdh* and *Trf* (n = 4–5 per genotype).
(PDF)

Figure S2 **Control stainings of tissues.** (**A**) No blue colour precipitate was visible in neonatal brain from wild-type or single transgenic *Cre*/+; +/+ or +/+; +/*XLlacZGT* mice. Shown is a XGal preparation of a wild-type whole-mount brain, cut sagittally along the midline before incubation in staining solution. (**B**) XGal preparation of a neonatal thoracic spine / intercostal muscle tissue sample of a *+*/+; +/*XLlacZGT* mouse. (**C**), (**D**) Immunohistochemistry of coronal sections of adult brain from wild-type (C) and *Gnasxl*-deficient (*Gnasxl* ^m+/p−^) (D) mice using the anti-XLαs antibody. The hypothalamus and amygdala are shown. Both sections were stained in the same experiment; lack of DAB/Ni precipitate in the *Gnasxl* knock-out sample (D) confirms the specificity of the antibody. (**E**), (**F**) Immunohistochemistry for XLαs on neonatal (P2) skeletal muscle sections from wild-type (E) and *Gnasxl* ^m+/p−^ mice (F). The comparatively low affinity/avidity of the XLαs antibody is sufficient for detection of expression in blood vessel, but did not produce a signal in skeletal muscle cells. This is in line with the relatively higher expression levels of *Gnasxl* in blood vessels as compared to skeletal muscle cells, which was evident from XGal stained sections of gene trap tissue (see Fig. 7B). (**G**), (**H**) Immunohistochemistry for XLαs on neonatal (P2) tongue muscle sections from wild-type (G) and *Gnasxl* ^m+/p−^ mice (H) confirms faithful reporter gene expression after gene trap activation (see Fig. 7I). (**I**) Neonatal (P1) wild-type pituitary whole-mount XGal control staining. (**J**) Kidney from an adult +/+; +/*XLlacZGT* control mouse (inactive gene trap) shows background β-Galactosidase like activity. The tissue was cut longitudinally before incubation in XGal solution. Amy – amygdala, PVH – paraventricular hypothalamic nucleus, BV – blood vessel.
(PDF)

Figure S3 **Expression and co-localisation of** ***Gnasxl*** **in the hypothalamus.** (**A**) Expression of *Gnasxl* in the hypothalamic arcuate nucleus at postnatal day 4. An *in situ* hybridisation of a sagittal brain section with a Digoxigenin-labelled RNA probe is shown. (**B**) XLαs expression in A12 dopaminergic neurons of the arcuate nucleus of adult mice. (B i) Overview; XLαs in green; Tyrosine hydroxylase (TH) in red. White arrow = A12 dopamine neuron group of the arcuate nucleus. Red arrow = A13 dopaminergic cells of the zona incerta (no co-localisation). (B ii) Higher magnification showing co-localisation in a portion of arcuate neurons. (B iii) ∼65% of TH positive neurons in the A12 dopaminergic cell group showed XLαs expression (n = 2).
(PDF)

Figure S4 **Plasma Ghrelin levels of** ***Gnasxl*** **^m+/p−^ mice are elevated.** Plasma Ghrelin levels (active Ghrelin and total Ghrelin) of *Gnasxl*-deficient (Plagge et al., 2004) adult females (n = 13) and their wild-type littermates (n = 15) under *ad libitum* fed normal chow diet conditions. Means ± SEM; ** p = 0.003 (active Ghrelin) and p<0.0001 (total Ghrelin) versus WT.
(PDF)

Figure S5 **Simplified scheme of the mTOR1-S6K nutrient sensing kinase pathway in the context of InsR and LepR signalling.** Both receptors can activate mTOR1-S6K via their IRS-PI3K-Akt signalling branch. Additional intracellular nutrient sensing mechanisms further influence mTOR1-S6K activity (not shown). In a negative feedback loop p70S6K1 phosphorylates and inhibits IRS1. Receptors are shaded as grey boxes, kinases as brown boxes. Phosphorylation of the S6K1 substrate S6 serves as a histological marker for its activity. (modified from Polak, P. and Hall, M. N., Curr. Op. Cell Biol. 21: 209–218 (2009); and Howell, J. J. and Manning, B. D., Trends Endocrinol. Metab., 22: 94–102 (2011)).
(PDF)

Figure S6 **XGal staining of (A) neonatal and (B) adult adrenal glands of** ***CMV-Cre*** **/+; +/** ***XLlacZGT*** **mice.** Tissues were cut transverse and stained as whole-mounts over night. Blue colour precipitate formed specifically in the adrenal medulla.
(PDF)

Table S1 **Sequences of oligonucleotides used for genotyping, RT-PCR and qRT-PCR in** **Figure 2** **and Figure S1.**
(PDF)

Table S2 **Summary of developmental changes in the brain expression pattern of** ***Gnasxl*** **.** Data are collated from XLαs/XLN1 immunohistochemistry, *XLlacZGT* gene trap fusion protein expression and *in situ* hybridisation studies in this manuscript and from Plagge A., et al., Nat. Genet. 36: 818–826 (2004). For abbreviations see main text and figure legends.
(PDF)
