# Supplementary material for: Postnatal Changes in the Expression Pattern of the Imprinted Signalling Protein XLαs Underlie the Changing Phenotype of Deficient Mice
Source: PLoS One. 2012 Jan 11;7(1):e29753. doi: 10.1371/journal.pone.0029753 (PMC3256176; doi:10.1371/journal.pone.0029753)
Supplement: Figure S1 — Analysis of Gnasxl transcript levels by qRT-PCR. RNA from neonatal brain of wild-type, Cre/+; +/XLlacZGT (inverted, active gene trap) and +/+; +/XLlacZGT (inactive gene trap) littermates was analysed using primers specific for Gnasxl exon 1. Expression levels were normalised to the housekeeping genes Gapdh and Trf (n = 4–5 per genotype). (PDF) [file pone.0029753.s001.pdf]

Figure S1

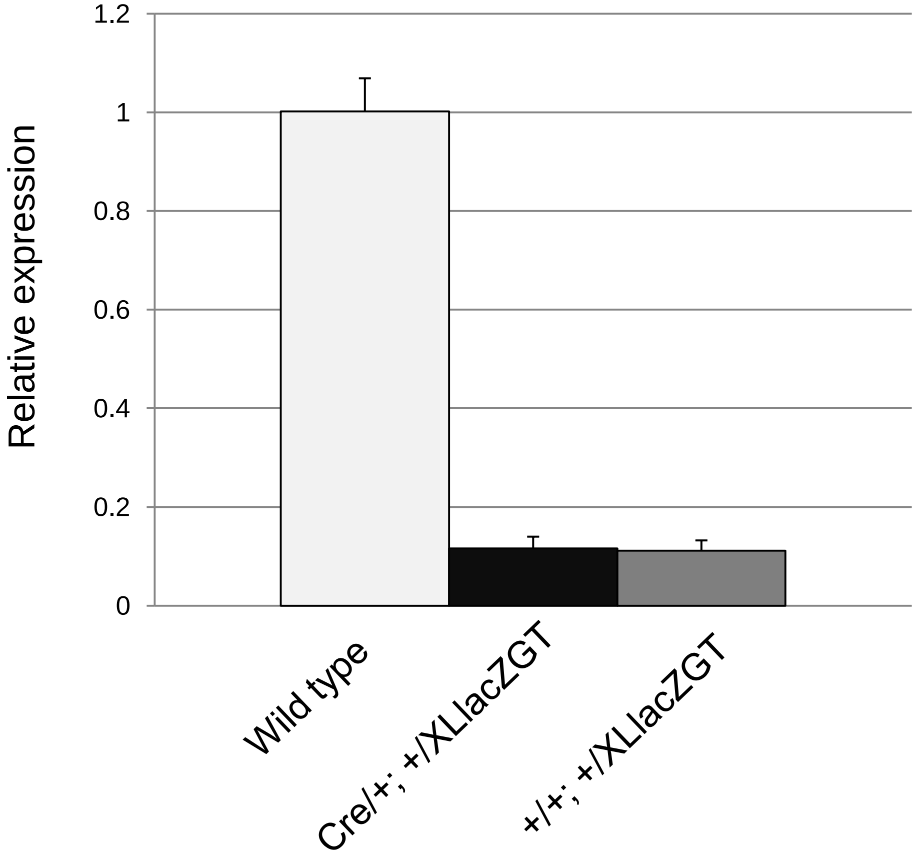

Figure S1. Analysis of *Gnasxl* transcript levels by qRT-PCR. RNA from neonatal brain of wild type, *Cre*/+; +/*XLlacZGT* (inverted, active gene trap) and +/+; +/*XLlacZGT* (inactive gene trap) littermates was analysed using primers specific for *Gnasxl* exon 1. Expression levels were normalised to the housekeeping genes *Gapdh* and *Trf* (n = 4 – 5 per genotype).
