# Supplementary material for: Postnatal Changes in the Expression Pattern of the Imprinted Signalling Protein XLαs Underlie the Changing Phenotype of Deficient Mice
Source: PLoS One. 2012 Jan 11;7(1):e29753. doi: 10.1371/journal.pone.0029753 (PMC3256176; doi:10.1371/journal.pone.0029753)
Supplement: Figure S2 — Control stainings of tissues. (A) No blue colour precipitate was visible in neonatal brain from wild-type or single transgenic Cre/+; +/+ or +/+; +/XLlacZGT mice. Shown is a XGal preparation of a wild-type whole-mount brain, cut sagittally along the midline before incubation in staining solution. (B) XGal preparation of a neonatal thoracic spine / intercostal muscle tissue sample of a +/+; +/XLlacZGT mouse. (C), (D) Immunohistochemistry of coronal sections of adult brain from wild-type (C) and Gnasxl-deficient (Gnasxl m+/p−) (D) mice using the anti-XLαs antibody. The hypothalamus and amygdala are shown. Both sections were stained in the same experiment; lack of DAB/Ni precipitate in the Gnasxl knock-out sample (D) confirms the specificity of the antibody. (E), (F) Immunohistochemistry for XLαs on neonatal (P2) skeletal muscle sections from wild-type (E) and Gnasxl m+/p− mice (F). The comparatively low affinity/avidity of the XLαs antibody is sufficient for detection of expression in blood vessel, but did not produce a signal in skeletal muscle cells. This is in line with the relatively higher expression levels of Gnasxl in blood vessels as compared to skeletal muscle cells, which was evident from XGal stained sections of gene trap tissue (see Fig. 7B). (G), (H) Immunohistochemistry for XLαs on neonatal (P2) tongue muscle sections from wild-type (G) and Gnasxl m+/p− mice (H) confirms faithful reporter gene expression after gene trap activation (see Fig. 7I). (I) Neonatal (P1) wild-type pituitary whole-mount XGal control staining. (J) Kidney from an adult +/+; +/XLlacZGT control mouse (inactive gene trap) shows background β-Galactosidase like activity. The tissue was cut longitudinally before incubation in XGal solution. Amy – amygdala, PVH – paraventricular hypothalamic nucleus, BV – blood vessel. (PDF) [file pone.0029753.s002.pdf]

Figure S2

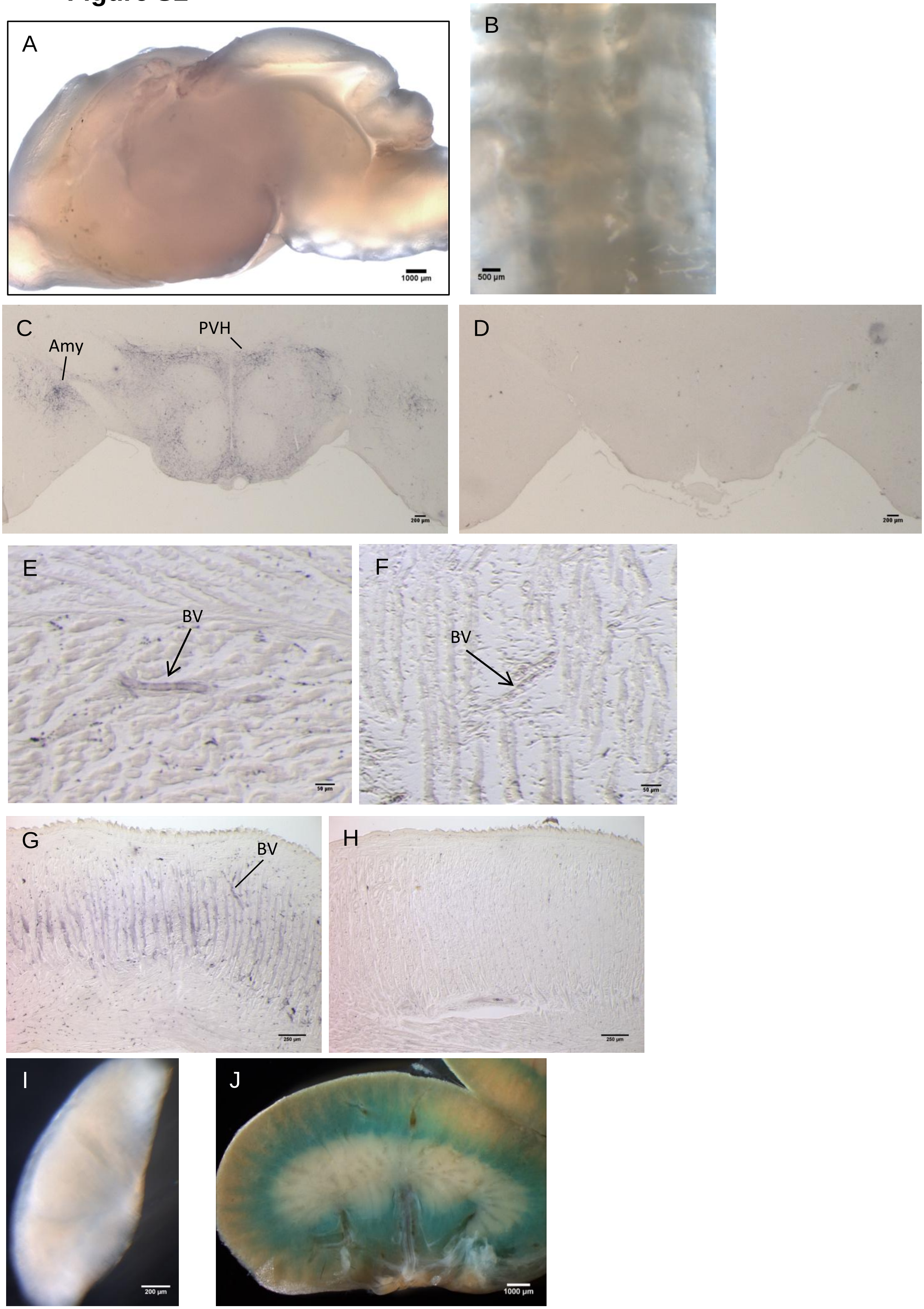
A
1000 µm
B
500 µm
C
Amy
PVH
200 µm
D
200 µm
E
BV
50 µm
F
BV
50 µm
G
BV
250 µm
H
250 µm
I
200 µm
J
1000 µm

Figure S2. Control stainings of tissues. (A) No blue colour precipitate was visible in neonatal brain from wild-type or single transgenic *Cre/+; +/+* or +/+; +/*XLlacZGT* mice. Shown is a XGal preparation of a wild-type whole-mount brain, cut sagittally along the midline before incubation in staining solution**.** **(B)** XGal preparation of a neonatal thoracic spine / intercostal muscle tissue sample of a *+/+; +/XLlacZGT* mouse. **(C), (D)** Immunohistochemistry of coronal sections of adult brain from wild-type (C) and *Gnasxl* deficient (*Gnasxl*$^{m+/p-}$) (D) mice using the anti-XLαs antibody. The hypothalamus and amygdala are shown. Both sections were stained in the same experiment; lack of DAB/Ni precipitate in the *Gnasxl* knock-out sample (D) confirms the specificity of the antibody. **(E), (F)** Immunohistochemistry for XLαs on neonatal (P2) skeletal muscle sections from wild-type (E) and *Gnasxl*$^{m+/p-}$ mice (F). The comparatively low affinity/avidity of the XLαs antibody is sufficient for detection of expression in blood vessel, but did not produce a signal in skeletal muscle cells. This is in line with the relatively higher expression levels of *Gnasxl* in blood vessels as compared to skeletal muscle cells, which was evident from XGal stained sections of gene trap tissue (see Fig. 7B). **(G), (H)** Immunohistochemistry for XLαs on neonatal (P2) tongue muscle sections from wild-type (G) and *Gnasxl*$^{m+/p-}$ mice (H) confirms faithful reporter gene expression after gene trap activation (see Fig. 7I). **(I)** Neonatal (P1) wild-type pituitary whole-mount XGal control staining. **(J)** Kidney from an adult +/+; +/*XLlacZGT* control mouse (inactive gene trap) shows background β-Galactosidase like activity. The tissue was cut longitudinally before incubation in XGal solution. Amy – amygdala, PVH – paraventricular hypothalamic nucleus, BV – blood vessel.
