# Supplementary material for: Postnatal Changes in the Expression Pattern of the Imprinted Signalling Protein XLαs Underlie the Changing Phenotype of Deficient Mice
Source: PLoS One. 2012 Jan 11;7(1):e29753. doi: 10.1371/journal.pone.0029753 (PMC3256176; doi:10.1371/journal.pone.0029753)
Supplement: Figure S3 — Expression and co-localisation of Gnasxl in the hypothalamus. (A) Expression of Gnasxl in the hypothalamic arcuate nucleus at postnatal day 4. An in situ hybridisation of a sagittal brain section with a Digoxigenin-labelled RNA probe is shown. (B) XLαs expression in A12 dopaminergic neurons of the arcuate nucleus of adult mice. (B i) Overview; XLαs in green; Tyrosine hydroxylase (TH) in red. White arrow = A12 dopamine neuron group of the arcuate nucleus. Red arrow = A13 dopaminergic cells of the zona incerta (no co-localisation). (B ii) Higher magnification showing co-localisation in a portion of arcuate neurons. (B iii) ∼65% of TH positive neurons in the A12 dopaminergic cell group showed XLαs expression (n = 2). (PDF) [file pone.0029753.s003.pdf]

Figure S3

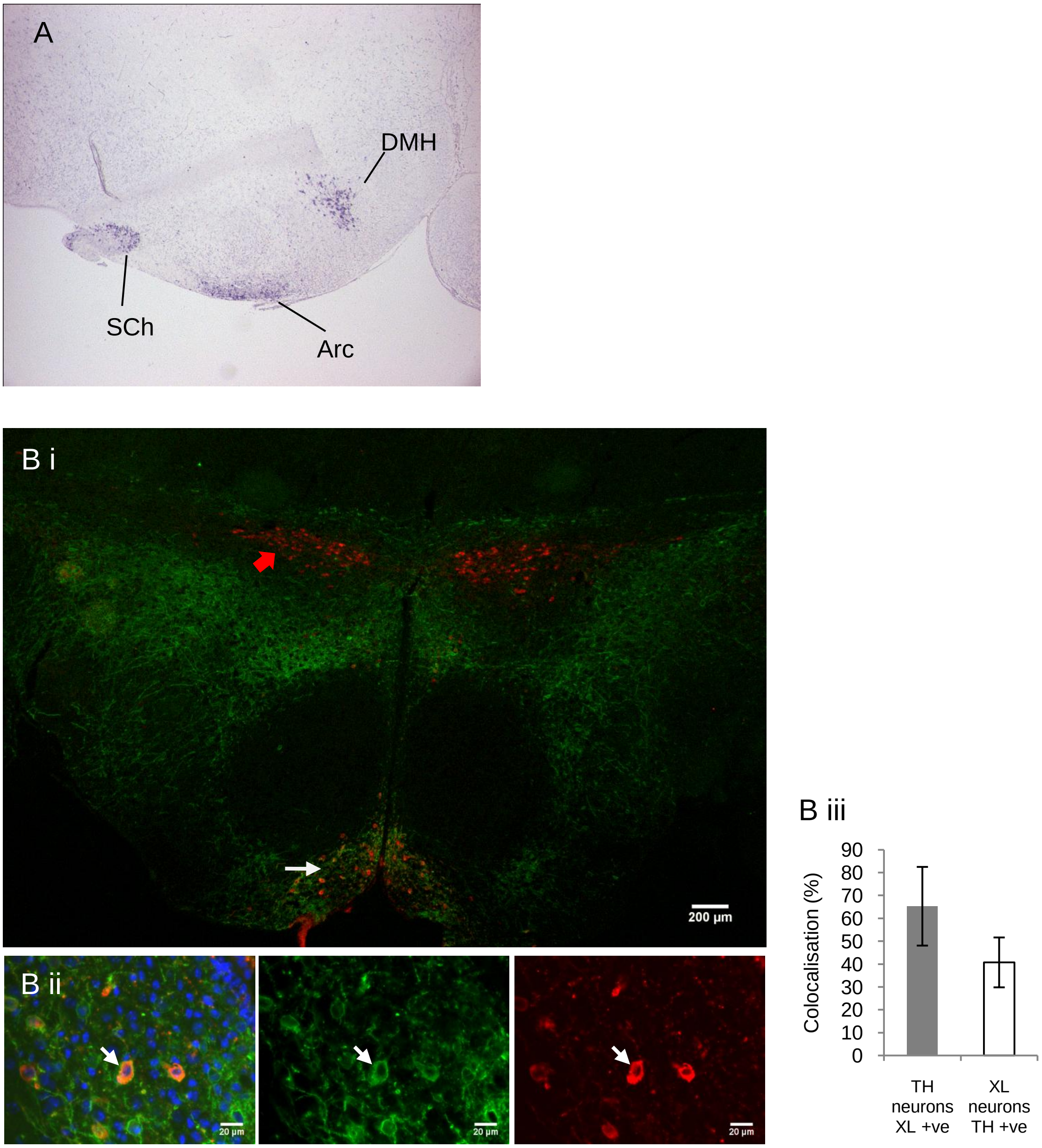

Figure S3. Expression and co-localisation of *Gnasxl* in the hypothalamus. (A) Expression of *Gnasxl* in the hypothalamic arcuate nucleus at postnatal day 4. An *in situ* hybridisation of a sagittal brain section with a Digoxigenin-labelled RNA probe is shown. **(B)** XLαs expression in A12 dopaminergic neurons of the arcuate nucleus of adult mice. **(B i)** Overview; XLαs in green; Tyrosine hydroxylase (TH) in red. White arrow = A12 dopamine neuron group of the arcuate nucleus. Red arrow = A13 dopaminergic cells of the zona incerta (no co-localisation). **(B ii)** Higher magnification showing co-localisation in a portion of arcuate neurons. **(B iii)** ~65 % of TH positive neurons in the A12 dopaminergic cell group showed XLαs expression (n =2).
