# Supplementary material for: Postnatal Changes in the Expression Pattern of the Imprinted Signalling Protein XLαs Underlie the Changing Phenotype of Deficient Mice
Source: PLoS One. 2012 Jan 11;7(1):e29753. doi: 10.1371/journal.pone.0029753 (PMC3256176; doi:10.1371/journal.pone.0029753)
Supplement: Figure S4 — Plasma Ghrelin levels of Gnasxl m+/p− mice are elevated. Plasma Ghrelin levels (active Ghrelin and total Ghrelin) of Gnasxl-deficient (Plagge et al., 2004) adult females (n = 13) and their wild-type littermates (n = 15) under ad libitum fed normal chow diet conditions. Means ± SEM; ** p = 0.003 (active Ghrelin) and p<0.0001 (total Ghrelin) versus WT. (PDF) [file pone.0029753.s004.pdf]

Figure S4

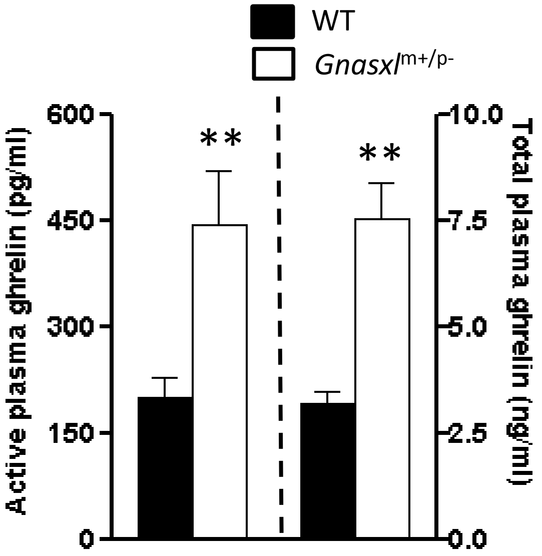

Figure S4. Plasma Ghrelin levels of *Gnasxl*$^{m+/p-}$ mice are elevated. Plasma Ghrelin levels (active Ghrelin and total Ghrelin) of *Gnasxl* deficient (Plagge et al., 2004) adult females (n=13) and their wild-type littermates (n=15) under *ad libitum* fed normal chow diet conditions. Means $\pm$ SEM; ** p = 0.003 (active Ghrelin) and p<0.0001 (total Ghrelin) versus WT.
