# Supplementary material for: Postnatal Changes in the Expression Pattern of the Imprinted Signalling Protein XLαs Underlie the Changing Phenotype of Deficient Mice
Source: PLoS One. 2012 Jan 11;7(1):e29753. doi: 10.1371/journal.pone.0029753 (PMC3256176; doi:10.1371/journal.pone.0029753)
Supplement: Figure S5 — Simplified scheme of the mTOR1-S6K nutrient sensing kinase pathway in the context of InsR and LepR signalling. Both receptors can activate mTOR1-S6K via their IRS-PI3K-Akt signalling branch. Additional intracellular nutrient sensing mechanisms further influence mTOR1-S6K activity (not shown). In a negative feedback loop p70S6K1 phosphorylates and inhibits IRS1. Receptors are shaded as grey boxes, kinases as brown boxes. Phosphorylation of the S6K1 substrate S6 serves as a histological marker for its activity. (modified from Polak, P. and Hall, M. N., Curr. Op. Cell Biol. 21: 209–218 (2009); and Howell, J. J. and Manning, B. D., Trends Endocrinol. Metab., 22: 94–102 (2011)). (PDF) [file pone.0029753.s005.pdf]

Figure S5

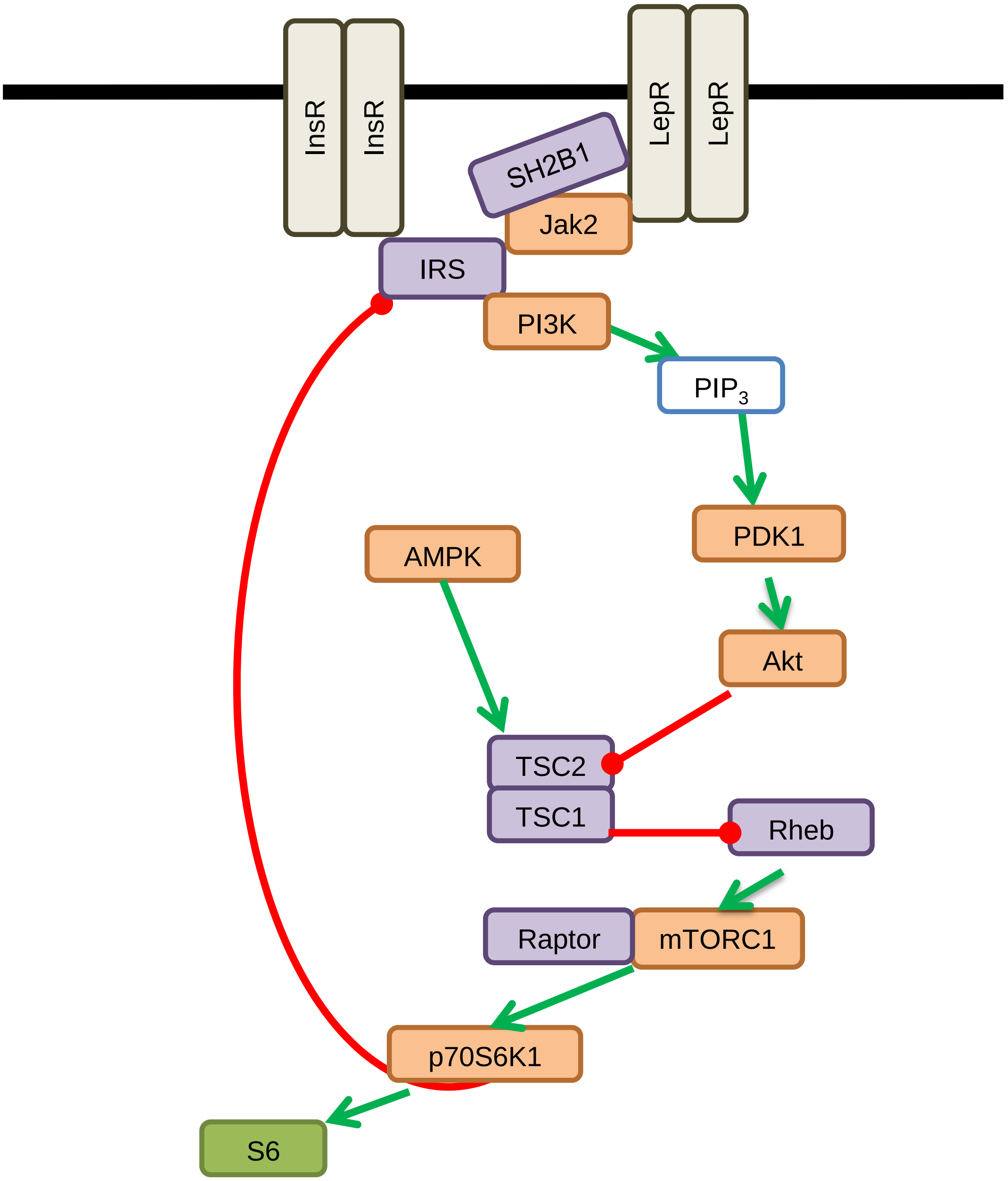

Figure S5. Simplified scheme of the mTOR-S6K nutrient sensing kinase pathway in the context of InsR and LepR signaling. Both receptors can activate mTOR-S6K via their IRS-PI3K-Akt signaling branch. Additional intracellular nutrient sensing mechanisms further influence mTOR-S6K activity (not shown). In a negative feedback loop p70S6K1 phosphorylates and inhibits IRS1. Receptors are shaded as grey boxes, kinases as brown boxes. Phosphorylation of the S6K1 substrate S6 serves as a histological marker for its activity.
(modified from Polak, P. and Hall, M. N., Curr. Op. Cell Biol. 21: 209-218 (2009); and Howell, J. J. and Manning, B. D., Trends Endocrinol. Metab., 22: 94-102 (2011)).
