# Supplementary material for: Postnatal Changes in the Expression Pattern of the Imprinted Signalling Protein XLαs Underlie the Changing Phenotype of Deficient Mice
Source: PLoS One. 2012 Jan 11;7(1):e29753. doi: 10.1371/journal.pone.0029753 (PMC3256176; doi:10.1371/journal.pone.0029753)
Supplement: Figure S6 — XGal staining of (A) neonatal and (B) adult adrenal glands of CMV-Cre /+; +/ XLlacZGT mice. Tissues were cut transverse and stained as whole-mounts over night. Blue colour precipitate formed specifically in the adrenal medulla. (PDF) [file pone.0029753.s006.pdf]

Figure S6

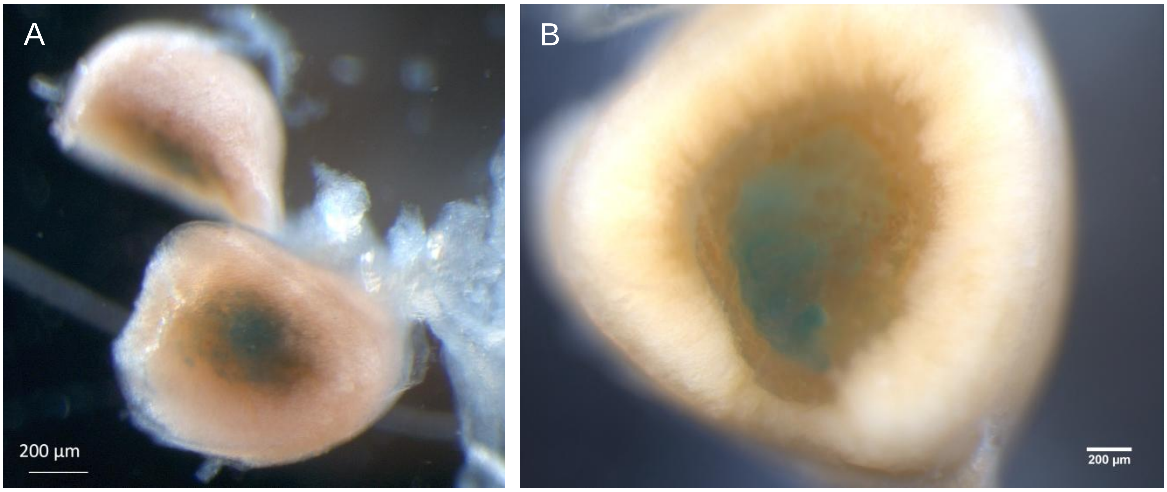

Figure S6. XGal staining of (A) neonatal and (B) adult adrenal glands of *CMV-Cre*/+; +/*XLlacZGT* mice. Tissues were cut transverse and stained as whole-mounts over night. Blue colour precipitate formed specifically in the adrenal medulla.
