# Supplementary material for: Postnatal Changes in the Expression Pattern of the Imprinted Signalling Protein XLαs Underlie the Changing Phenotype of Deficient Mice
Source: PLoS One. 2012 Jan 11;7(1):e29753. doi: 10.1371/journal.pone.0029753 (PMC3256176; doi:10.1371/journal.pone.0029753)
Supplement: Table S1 — Sequences of oligonucleotides used for genotyping, RT-PCR and qRT-PCR in Figure 2 and Figure S1. (PDF) [file pone.0029753.s007.pdf]

Table S1

F1<br>(Qxl-F1) (Figure 2A, C, E)	5’-TAAGAAACGCAGCAAGCTCA-3’
F2<br>(5’GTFlp F1) (Figure 2A, C)	5’-TAGGTAATGCGGCAGCTTCT-3’
R1<br>(3GTFlp F1) (Figure 2A, C)	5’-CTCGCTATTATTCCAACCGC-3’
R2<br>(5GTFlp R1) (Figure 2A, C)	5’-CCTCTTCGCTATTACGCCAG-3’
R3<br>(3GTFlp R1) (Figure 2A, C)	5’-AGTTCACAAGGCTATCCCCA-3’
ex5-R<br>(exon5-R1) (Figure 2E)	5’-GAGGACTGTAGCCATCATCT-3’
N1-R<br>(XLN1-R6) (Figure 2E)	5’-TCTAGTGGGGGTAATAGACT-3’
Gxll-F1<br>(Gnasxl qPCR) (Figure S1)	5’-GGATCCTATGGAGGAGAGGC-3’
Gxll-R1<br>(Gnasxl qPCR) (Figure S1)	5’-TCCAGTTGCTTGTCGATGAG-3’

Table S1. Sequences of oligonucleotides used for genotyping, RT-PCR and qRT-PCR in Figure 2 and Figure S1.
