# Supplementary material for: Postnatal Changes in the Expression Pattern of the Imprinted Signalling Protein XLαs Underlie the Changing Phenotype of Deficient Mice
Source: PLoS One. 2012 Jan 11;7(1):e29753. doi: 10.1371/journal.pone.0029753 (PMC3256176; doi:10.1371/journal.pone.0029753)
Supplement: Table S2 — Summary of developmental changes in the brain expression pattern of Gnasxl . Data are collated from XLαs/XLN1 immunohistochemistry, XLlacZGT gene trap fusion protein expression and in situ hybridisation studies in this manuscript and from Plagge A., et al., Nat. Genet. 36: 818–826 (2004). For abbreviations see main text and figure legends. (PDF) [file pone.0029753.s008.pdf]

Table S2:

**Brain region:**	**Expression at neonatal stages only**	**Expression at adult stages only**	**Expression at neonatal and adult stages**
**Midbrain and brainstem**	LDTg	NTS	Orofacial motor nuclei (12N, 7N, 5N, expression levels decrease in adults)<br><br>Gi/MRt, Amb, ROb, RPa,<br><br>PTg, SubC, LC, A7
**Spinal cord**			IML<br><br>Ventral motoneurons (expression levels decrease in adults)
**Hypothalamus, Preoptic areas, Amygdala**		Amygdala (medial part)	DMH, LH, PVH, Arc, SCh, PreOp including subregions of the bed nucleus of the stria terminalis, lateral septal nucleus and A14 dopaminergic neurons;<br><br>(expression levels in all areas increase towards adulthood)

Table S2: Summary of developmental changes in the brain expression pattern of *Gnasxl.* Data are collated from XLαs immunohistochemistry, *XLlacZGT* gene trap fusion protein expression and *in situ* hybridisation studies in this manuscript and from Plagge A., et al., Nat. Genet. 36: 818-826 (2004). For abbreviations see main text and figure legends.
